# Research trends in depression associated with gynecologic cancers from 2006 to 2025: a multidatabase bibliometric and visual analysis

**DOI:** 10.3389/fonc.2026.1910076

**Published:** 2026-08-12

**Authors:** Xu Feng, Lu Chen, Rubing Wang, Ziheng Wang, Liqian Feng, Yongxin Zhou, Jinying Zhao, Fuchun Wang

**Affiliations:** College of Acupuncture and Tuina, Changchun University of Chinese Medicine, Changchun, China

**Keywords:** bibliometrics, depression, gynecological cancers, quality of life, visualization

## Abstract

**Background:**

Gynecologic malignancies are an important cause of illness among women. Depressive symptoms are common throughout diagnosis, treatment, and survivorship. However, bibliometric evidence concerning this topic remains scarce. This bibliometric analysis characterizes research trends and identifies major research hotspots in the literature on depression associated with gynecologic cancer, thereby informing future research and clinical practice.

**Methods:**

Relevant publications issued from January 1, 2006, to December 31, 2025, were identified through WOSCC, PubMed, and Scopus. The records were examined and mapped with VOSviewer, CiteSpace, and bibliometrix.

**Results:**

The final analysis comprised 734 publications. Research output on depression in patients with gynecologic cancer generally increased from 2006 to 2025. The USA accounted for the greatest share of the literature, and the University of Texas MD Anderson Cancer Center contributed the most papers among institutions. Sood, Anil K. had the most prolific author, whereas the publications by Lutgendorf, Susan K. had the highest average citation count. Among the journals analyzed, *Gynecologic Oncology* ranked first in terms of both publication output and total citations. The most frequent keywords were “quality of life”, “depression”, “women”, “ovarian cancer”, “cervical cancer”. Keywords with citation bursts continuing through 2025 included “cohort analysis”, “retrospective study”, “patient-reported outcomes”, “nausea”, “multicenter study”, “uterus cancer” and “sleep disturbance”.

**Conclusions:**

Depression associated with gynecologic cancers has received increasing attention, with growing emphasis on patients’ quality of life. Future research may increasingly focus on the treatment and supportive care of depression in patients with gynecologic cancers.

## 1 Introduction

Gynecologic malignancies comprise several types, including cervical, ovarian, uterine, vulvar, and vaginal cancers. Together, these malignancies remain an important women’s health concern worldwide (1). Cervical, endometrial, and ovarian cancers are the most frequently encountered gynecologic malignancies (2). According to GLOBOCAN 2020 estimates from the IARC, they accounted for 6.5%, 4.5%, and 3.4% of all new cancer cases among women worldwide, respectively (3). Their incidence rates have increased annually, accompanied by a trend toward younger age at onset (4, 5). Gynecologic cancers not only threaten patients’ health and survival but also impose a substantial economic burden on society. According to available statistics, ovarian cancer has caused an estimated US$70 billion in global economic losses (6). For women undergoing treatment for gynecologic cancer, improving quality of life may alleviate the physical and emotional effects of treatment and facilitate a return to occupational and social roles.

Patients with gynecologic cancer often experience depressive symptoms, anxiety, and sleep disturbances, which may reflect not only the burden of the disease but also the impact of treatment on body image, sexual function, and fertility (7). During the two years following diagnosis, women with cervical cancer were nearly four times as likely to receive a new diagnosis of depression as women in the general population (8). Post-treatment depression was reported in 12.7% of women with ovarian cancer (9), whereas 32.8% of endometrial cancer survivors reported depressive symptoms (10). Recent studies have provided a clearer picture of the psychological difficulties faced by women with gynecologic cancer. Longitudinal studies have shown that more severe depressive symptoms are generally accompanied by greater symptom burden, sleep disturbance, fatigue, and lower quality of life (11). Other studies have reported that financial stress and unmet supportive care needs are associated with different cancer types and disease stages (12). A 2026 meta-analysis specifically examined depression among women with cervical cancer, indicating that the related literature continues to expand (13).

Depression directly affects treatment outcomes in patients with gynecologic cancers and survivors. Depression and anxiety may influence drug responses by altering hypothalamic-pituitary-adrenal axis activity, immune regulation, and inflammatory signaling, thereby affecting treatment outcomes (14). Depression can also adversely affect patients’ quality of life (15). Depression can lead to persistent emotional dysregulation and impaired cognitive function, thereby adversely affecting learning, work, and social functioning (16). Patients with gynecologic cancers and major depressive disorder often develop suicidal ideation due to poor disease control, surgery-related physical disfigurement, family relationship difficulties, financial hardship, and inadequate social support (17). Clinical studies have shown that improving emotional states such as anxiety and depression has a positive effect on the regulation of antitumor immune responses (18). The interplay between depression and gynecologic cancers underscores the need to integrate routine psychological assessment and timely interventions for depression into cancer treatment and survivorship follow-up. These measures may help improve patients’ clinical outcomes.

Bibliometric analysis is a literature analysis method grounded in mathematical statistics that enables the systematic mapping and visualization of publications in a specific research field. This method helps reveal the developmental trajectory of a field, publication trends, journal-source structure, scientific collaboration networks, co-citation relationships, and the evolution of research themes. It can also identify influential authors, institutions, and research directions, thereby helping researchers understand the knowledge structure, research hotspots, and potential limitations of a field (19). Because the research questions in this study were not limited to publication output, we used bibliometric methods to analyze the development of the field, core contributors, collaboration patterns, citation links, and temporal changes in research themes (20–23). It should be noted that bibliometric analysis is not suitable for estimating disease prevalence, evaluating prognosis, or determining treatment effects; such questions are more appropriately addressed through systematic reviews and meta-analyses. Within these methodological boundaries, this study supplemented quantitative science mapping with structured thematic interpretation and cross-checked cluster results against representative publications to reduce the risk of making inferences based solely on software-generated labels (24, 25).

Several bibliometric studies have examined related topics. Zhao et al. mapped 6,479 WOSCC publications on quality of life or psychological impact in gynecologic cancer (26); Levin et al. examined country-income disparities in major gynecologic oncology journals (27); and Almobarak analyzed palliative care research in cervical cancer (28). However, none of these studies used depression as the principal inclusion concept across WOSCC, Scopus, and PubMed through 2025. Accordingly, the development and thematic evolution of depression research across different gynecologic cancers, as well as the links between clinical topics and collaboration networks, remain insufficiently characterized. Therefore, CiteSpace, VOSviewer, and the bibliometrix R package were used to conduct bibliometric and visualization analyses of relevant publications from January 1, 2006, to December 31, 2025. This study aimed to provide an innovative research perspective, establish a scientific foundation for future research, facilitate more precise and effective treatment strategies, and improve patients’ quality of life and clinical outcomes.

Accordingly, this study addressed the following four core questions: (1) How did publication output, geographic distribution, institutional composition, authorship patterns, collaboration networks, and publication-source patterns evolve from 2006 to 2025? (2) Which journals served as the principal publication sources, and which co-cited references formed the knowledge base of the field? (3) Which themes recurred across high-frequency keywords, co-cited references, keyword clusters, and keyword bursts? (4) What patterns of literature distribution and author productivity were observed through analyses based on Bradford’s and Lotka’s laws?

## 2 Materials and methods

### 2.1 Literature search and screening

Relevant records were retrieved from the Web of Science Core Collection (WOSCC), Scopus, and PubMed to ensure broad coverage of the literature. WOSCC is widely recognized for its high-quality and well-structured citation system (29). Scopus is one of the largest peer-reviewed literature databases worldwide, providing broad multidisciplinary coverage and systematically curated bibliographic information (30). PubMed is the most authoritative database in the biomedical field and serves as a crucial source for highly relevant medical literature.

Database-specific search strategies were developed using two consistent concept blocks: gynecologic neoplasms and depression. Terms within the two concept groups were linked with Boolean operators and database-specific truncation rules. Searches covered Topic in WOSCC, TITLE-ABS-KEY in Scopus, and Title/Abstract in PubMed. In WOSCC, a Topic search covers the title, abstract, author keywords, and Keywords Plus fields. Only articles and reviews published in English from January 1, 2006, through December 31, 2025, were considered. The 2025 cutoff was selected to minimize potential instability arising from the ongoing indexing of records published in early 2026. The final searches were conducted on May 16, 2026, and the complete search strategies are provided in **Supplementary Table 1**.

**Figure 1:**
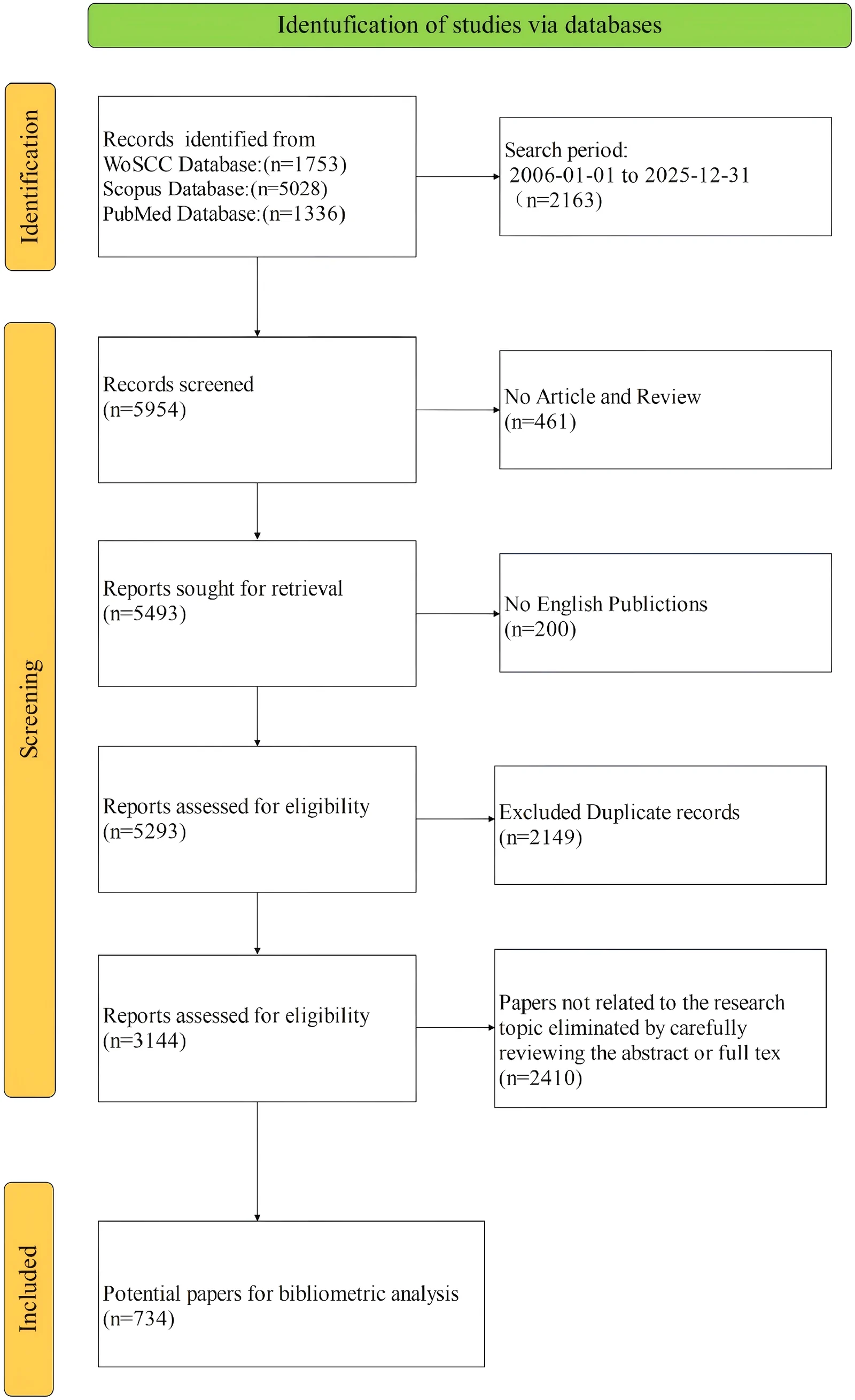
Flowchart for the research’s search process.

The database searches identified 8,117 records, including 1,753 from WOSCC, 5,028 from Scopus, and 1,336 from PubMed. After restricting the results to English-language articles and reviews published between January 1, 2006, and December 31, 2025, 5,293 records remained. We then removed 2,149 duplicates based on DOIs and other bibliographic information, leaving 3,144 records for screening. Complete bibliographic information, including cited references, was downloaded as plain-text or CSV files. Python 3.11 was used to convert the Scopus CSV and PubMed TXT files into plain text following the WOSCC structure (31). Data cleaning involved DOI-based duplicate removal, deletion of retracted publications and records with [Anonymous] in the author field, and removal of non-research entities such as the Egyptian Knowledge Bank (31). Different names referring to the same institution were also merged. XF and LC independently screened the titles and abstracts using the inclusion and exclusion criteria described below. They then reviewed the full texts of articles considered potentially eligible. Any disagreements were settled through discussion, and JZ adjudicated when needed. After excluding 2,410 records that did not meet the criteria, 734 publications were included in the final analysis. The study selection process is presented in **Figure 1**.

#### 2.1.1 Inclusion criteria

Studies were included if they met all of the following criteria: (1) the study population comprised women diagnosed with gynecologic cancer, including those undergoing treatment and cancer survivors; (2) depression, depressive symptoms, or depressive disorders were examined as a primary variable, outcome, exposure, or explicitly analyzed correlate; (3) the publication was an original research article or review published in English from 2006 through 2025; and (4) sufficient bibliographic metadata were available. All four criteria were required for inclusion; studies failing to meet any one criterion were excluded.

#### 2.1.2 Exclusion criteria

Records were excluded if they met any of the following criteria: (1) the study focused on benign gynecologic diseases, screening populations without a confirmed cancer diagnosis, non-gynecologic cancers for which relevant data could not be separately extracted, or anxiety or quality of life without an identifiable measure of depression; or (2) the record was an editorial, letter, conference abstract, protocol, news item; or (3) retracted publication; or (4) lacked identifiable authorship.

### 2.2 Data analysis

Data processing, analysis, and visualization were performed using CiteSpace 6.4.R1, VOSviewer 1.6.20, the bibliometrix package in R, Microsoft Excel 2021, and SCImago Graphica 1.0.55. These tools have complementary analytical functions. Specifically, bibliometrix was used for country-level analysis, Lotka’s law analysis, Bradford’s law analysis, thematic mapping, thematic evolution analysis, and trend topic analysis (32). VOSviewer was used to construct collaboration networks of research institutions, journals, and authors (33). CiteSpace was used for time-sliced co-citation analysis, keyword co-occurrence and clustering analysis, timeline visualization, and burst analysis (34, 35). Microsoft Excel and SCImago Graphica were used for chart organization, plotting, and presentation.

To reduce potential analytical bias arising from the use of a single software program and to improve the internal consistency of bibliometric interpretation, this study comprehensively compared the analytical outputs generated by multiple tools. The knowledge structure and temporal evolution of the field were interpreted through cross-validation across different science maps. This strategy was intended to make use of the complementary strengths of bibliometrix, VOSviewer, and CiteSpace in science mapping, collaboration network identification, and thematic evolution analysis, rather than relying on a single visualization result for inference.

Before the country-level analyses, geographic labels were standardized across databases. Records assigned to Taiwan were combined with China, whereas England, Scotland, Wales, and Northern Ireland were grouped under the United Kingdom. These rules were applied consistently to national publication counts, citation statistics, and collaboration networks.

#### 2.2.1 Bibliometric-law analysis

Source scattering and author productivity were examined using the “Bradford’s law” and “Lotka’s law” functions in bibliometrix in R. For the Bradford analysis, sources were ranked in descending order of publication count. Cumulative publication counts were regressed on the natural logarithm of source rank, and the ranked sources were divided into three zones, each containing approximately one-third of the publications. If n1, n2, and n3 denote the numbers of sources in the three zones, the software calculated the Bradford multiplier as k = [(n_2_/n_1_) + (n_3_/n_2_)]/2. For the Lotka analysis, ordinary least-squares regression was used to fit the proportion fn of authors with n publications on the log–log scale: log_10_ f(n) = log_10_ C − β log_10_ n. The software also reported Kolmogorov–Smirnov p values for the Bradford fit and for comparisons of the empirical Lotka distribution with the classical β=2 and fitted distributions. These p values were treated as descriptive fit diagnostics rather than formal inferential tests because the data were discrete and the fitted quantities were derived from the same dataset.

#### 2.2.2 Zipf-type keyword rank-frequency analysis

We used a Zipf-type keyword rank-frequency analysis to describe how keyword use was distributed between high-frequency themes and the long tail of less frequent terms. The analysis was based on the harmonized keyword-frequency table produced after keyword merging. Keywords were ranked from highest to lowest frequency, and the relationship between keyword frequency, f(r), and rank, r, was fitted on a log-log scale using the equation log10[f(r)] = a − s log10(r), where (s) denotes the Zipf-type exponent. This analysis was descriptive and was not used to test clinical significance or causal relationships.

## 3 Results

### 3.1 Trends in publications

**Figure 2:**
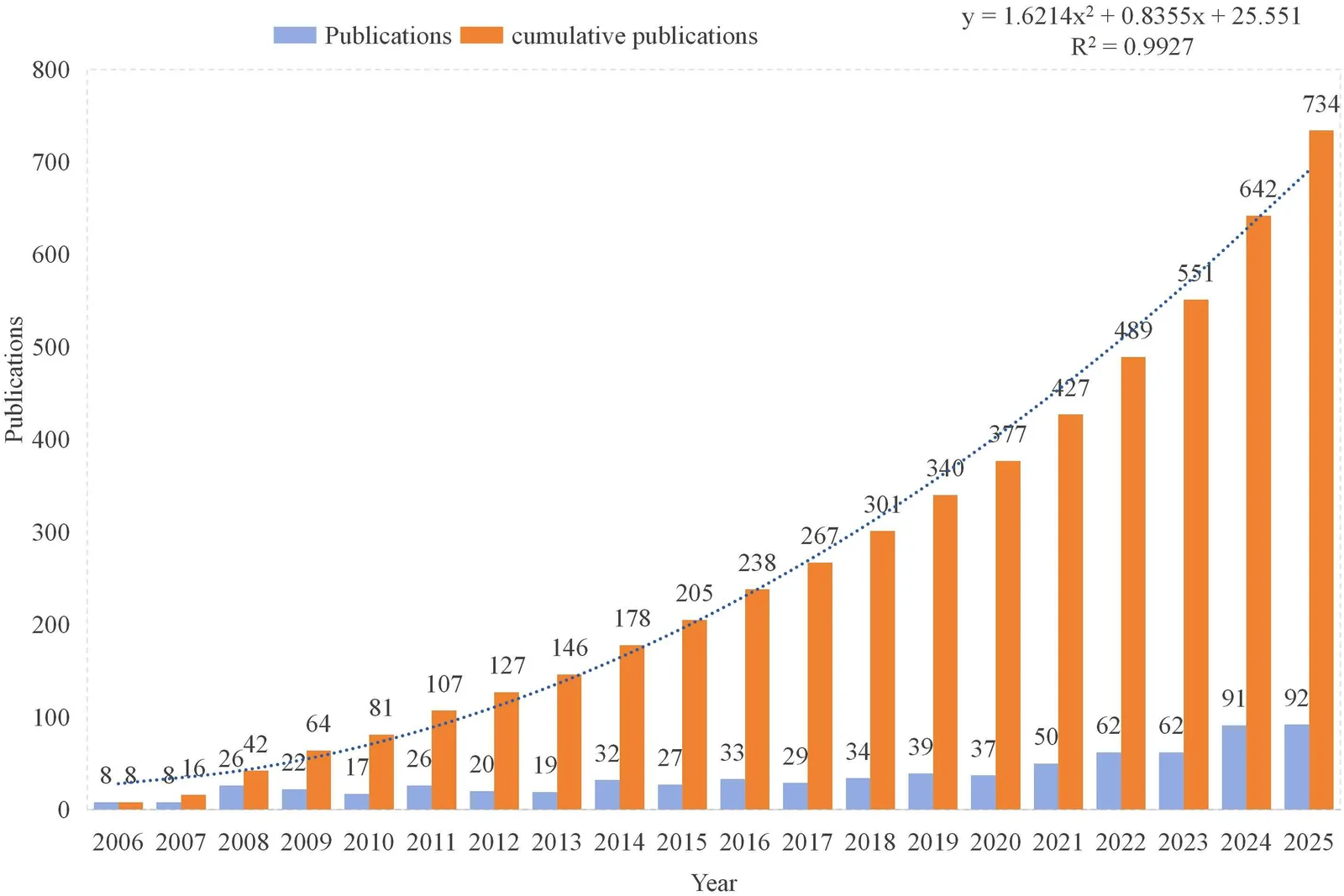
Annual trends in publication output.

The final analysis included 734 publications, comprising 672 original articles and 62 reviews. **Figure 2** shows the annual and cumulative publication counts. The annual number generally increased over time, and the linear fit yielded R^2^ = 0.9927. From 2021 to 2025, more than 50 papers were published each year.

The growth in publication volume provides context for the thematic findings reported below. The increase in annual publications after 2021 was accompanied by burst terms such as “patient-reported outcomes,” “sleep disturbance,” “cohort analysis,” “retrospective study,” and “multicenter study.” This pattern suggests that recent studies have built on earlier work on depression and quality of life in gynecologic cancer, while adding more detailed outcome reporting, symptom assessment, and observational study designs.

### 3.2 Analysis of countries or regions

**Figure 3:**
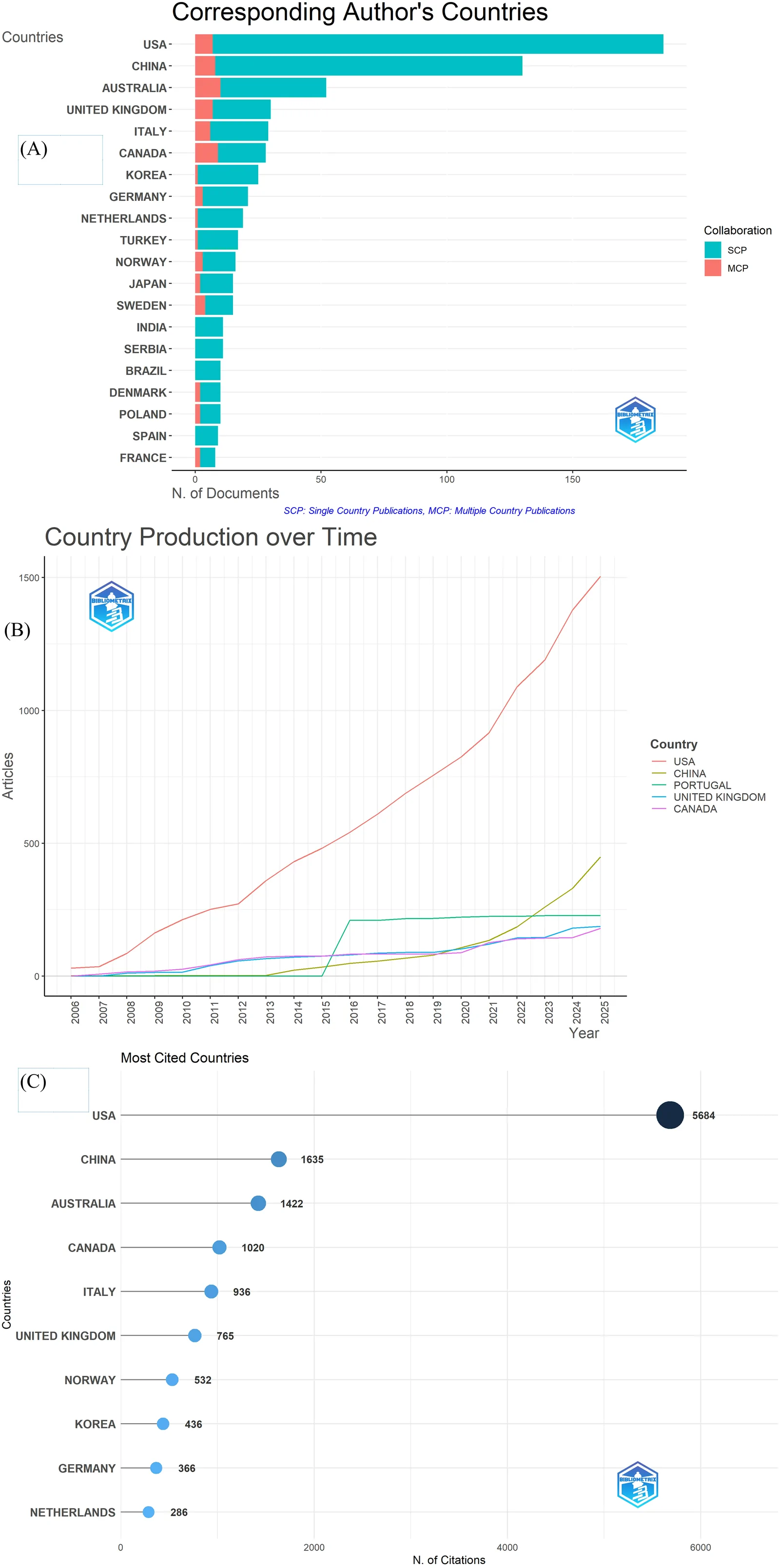
**(A)** Countries of corresponding authors; internationally collaborative publications are shown in orange. SCP, single-country publications; MCP, multiple-country publications. **(B)** Annual publication output of the five most productive countries. **(C)** Top 10 countries by total citations.

The study included 734 publications from 44 countries. As shown in **Table 1**, the USA published the most articles (n=186). China ranked second (n=130), followed by Australia (n=52). Notably, each country represented in an internationally coauthored publication was credited with one publication. Consequently, the sum of country-level publication counts exceeded the total number of included publications. **Figure 3A** shows limited international collaboration in research on depression associated with gynecologic cancers, with relatively few publications involving researchers from multiple countries. Interestingly, the USA, which ranked first in publication output, had an international collaboration rate of only 3.76%, whereas Canada had the highest rate at 32.14%. These findings indicate considerable scope for greater collaboration in research on depression associated with gynecologic cancers. The annual publication output of the five most productive countries was also analyzed. As shown in **Figure 3B**, the USA had the highest annual publication output and maintained its leading position from 2006 to 2025, demonstrating its substantial influence on the research landscape of this field. Interestingly, China did not begin to emerge in the field of depression associated with gynecologic cancers until 2021; however, its research output increased rapidly from 2021 to 2025. Among the 10 most frequently cited countries (**Figure 3C**), publications from the USA received the most citations (n=5,684). Next were China (n=1,635) and Australia (n=1,422). However, Canada had the highest average citation count (36.4). Notably, despite its relatively low publication output, Norway demonstrated strong research potential, with 532 cumulative citations and a mean of 33.2 citations per publication.

**Table 1:** The top 10 countries or regions ranked by publication number.

Rank	Country	Counts	% of 734	SCP	MCP	MCP%	Citations	Average citations
1	USA	186	25.34	179	7	3.76	5684	30.6
2	China	130	17.71	122	8	6.15	1635	12.6
3	Australia	52	7.08	42	10	19.23	1422	27.3
4	United Kingdom	30	4.08	23	7	23.33	765	25.5
5	Italy	29	3.95	23	6	20.68	936	32.3
6	Canada	28	3.81	19	9	32.14	1020	36.4
7	Korea	25	3.40	24	1	4	436	17.4
8	Germany	21	2.86	18	3	14.28	366	17.4
9	Netherlands	19	2.58	18	1	5.26	286	15.1
10	Turkey	17	2.31	16	1	5.88	267	15.7

Country-level analysis provides an important geographic perspective for understanding the thematic evolution of research on depression in gynecologic cancer. The USA ranked first in both publication output and total citations, indicating a strong research foundation and substantial academic influence in this field. By comparison, China showed rapid growth in publication output after 2021, reflecting increasing scholarly attention to this topic. Overall, the geographic distribution of research on depression in gynecologic cancer is gradually expanding, but collaboration networks among countries remain insufficiently connected. Future studies should therefore strengthen cross-regional, multicenter, and international collaboration.

### 3.3 Analysis of institutional outputs and cooperation

Between 2006 and 2025, 1,470 institutions contributed to research in this field. **Table 2** lists the 12 most productive institutions. The University of Texas MD Anderson Cancer Center published 22 papers. The University of Sydney and the University of Iowa published 17 and 16 papers, respectively. The University of Texas MD Anderson Cancer Center also received the most citations (n=803), indicating the broad recognition of its research contributions. The University of Queensland had the highest total link strength, indicating frequent collaboration with other institutions. More than half of the 12 leading institutions were based in the USA, showing its strong presence in this area.

**Table 2:** Top 12 institutions in the number of publications.

Rank	Institutions	Country	Documents	Citations	Total link strength
1	The University of Texas MD Anderson Cancer Center	USA	22	803	36
2	University of Sydney	Australia	17	418	47
3	University of Iowa	USA	16	729	35
4	University of California, Los Angeles	USA	13	420	31
5	Dana-Farber Cancer Institute	USA	13	395	18
6	The University of Queensland	Australia	11	244	49
7	University of Melbourne	Australia	11	195	37
8	Memorial Sloan Kettering Cancer Center	USA	11	544	30
9	China Medical University	China	11	225	1
10	Washington University in St. Louis	USA	10	307	31
11	University of California, San Francisco	USA	10	94	11
12	University of Oslo	Norway	10	254	3

**Figure 4:**
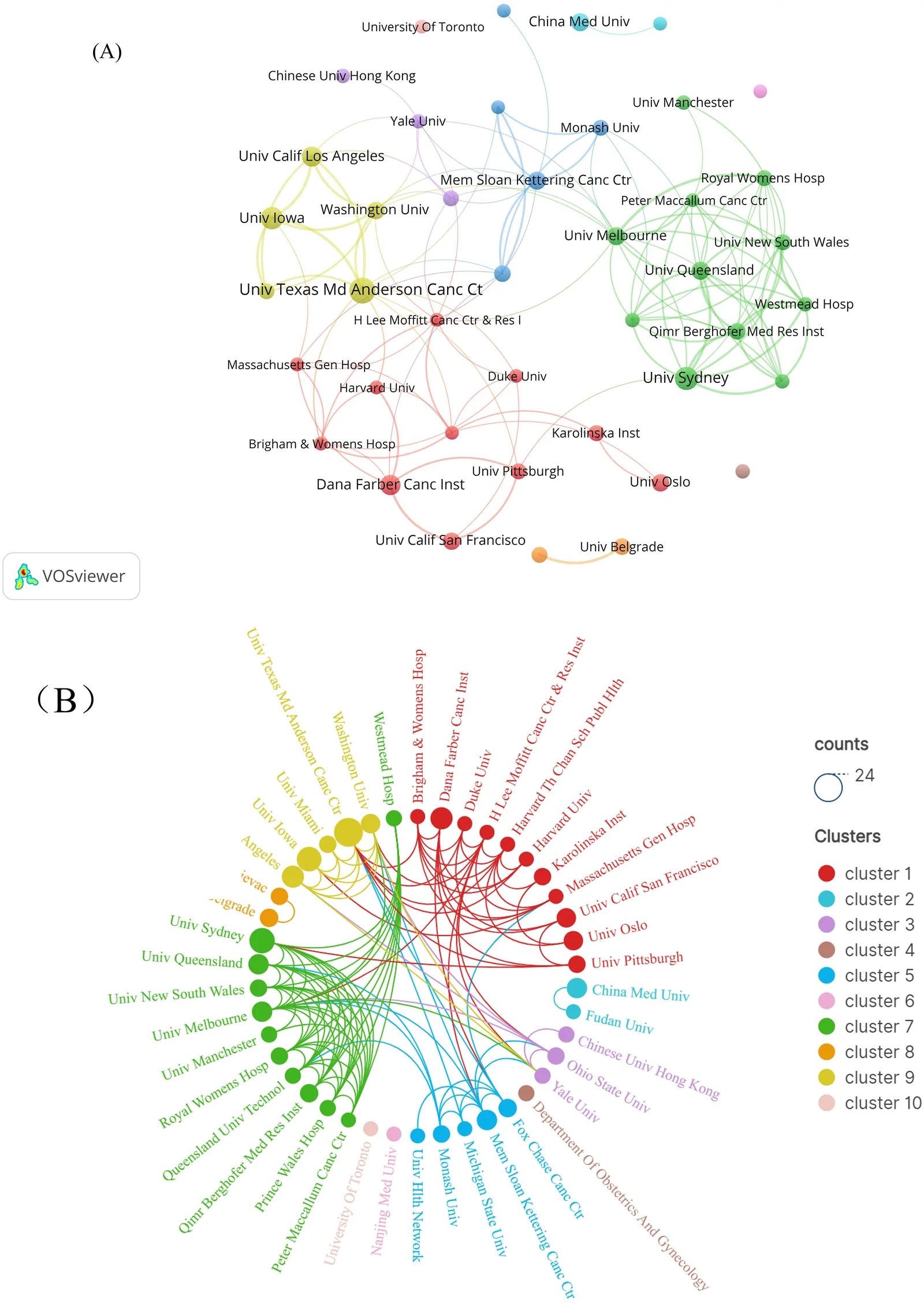
**(A)** Co-occurrence network diagram of the issuing institution; **(B)** chord chart of cooperation between institutions.

The institutional collaboration network included 42 institutions with at least six publications (**Figure 4A**). Their connections are shown in the chord diagram in **Figure 4B**. The network contained 10 clusters, including the University of Texas MD Anderson Cancer Center, the University of Sydney, and Dana-Farber Cancer Institute. However, collaboration between institutions was still limited and should be expanded.

Institutional collaboration analysis further complemented the country-level findings on geographic distribution. Institutions in the USA and Australia made relatively prominent contributions to research on depression in gynecologic cancer. However, the overall collaboration network remained dispersed, with limited connections among institutions. This insufficient collaboration may limit the integration of research questions, data resources, and methodological frameworks. It may also partly explain why the field has developed several relatively independent research directions, such as quality of life, symptom burden, sexual function, supportive care, and patient-reported outcomes, rather than more systematic and continuous research directions.

### 3.4 Analysis of authors

Research in this area involved 3,909 authors. According to Price’s Law (36, 37), the minimum number of publications required to qualify as a core author was calculated using the following formula:


$$M=0.749\times \sqrt{N_{max}}$$

**Figure 5:**
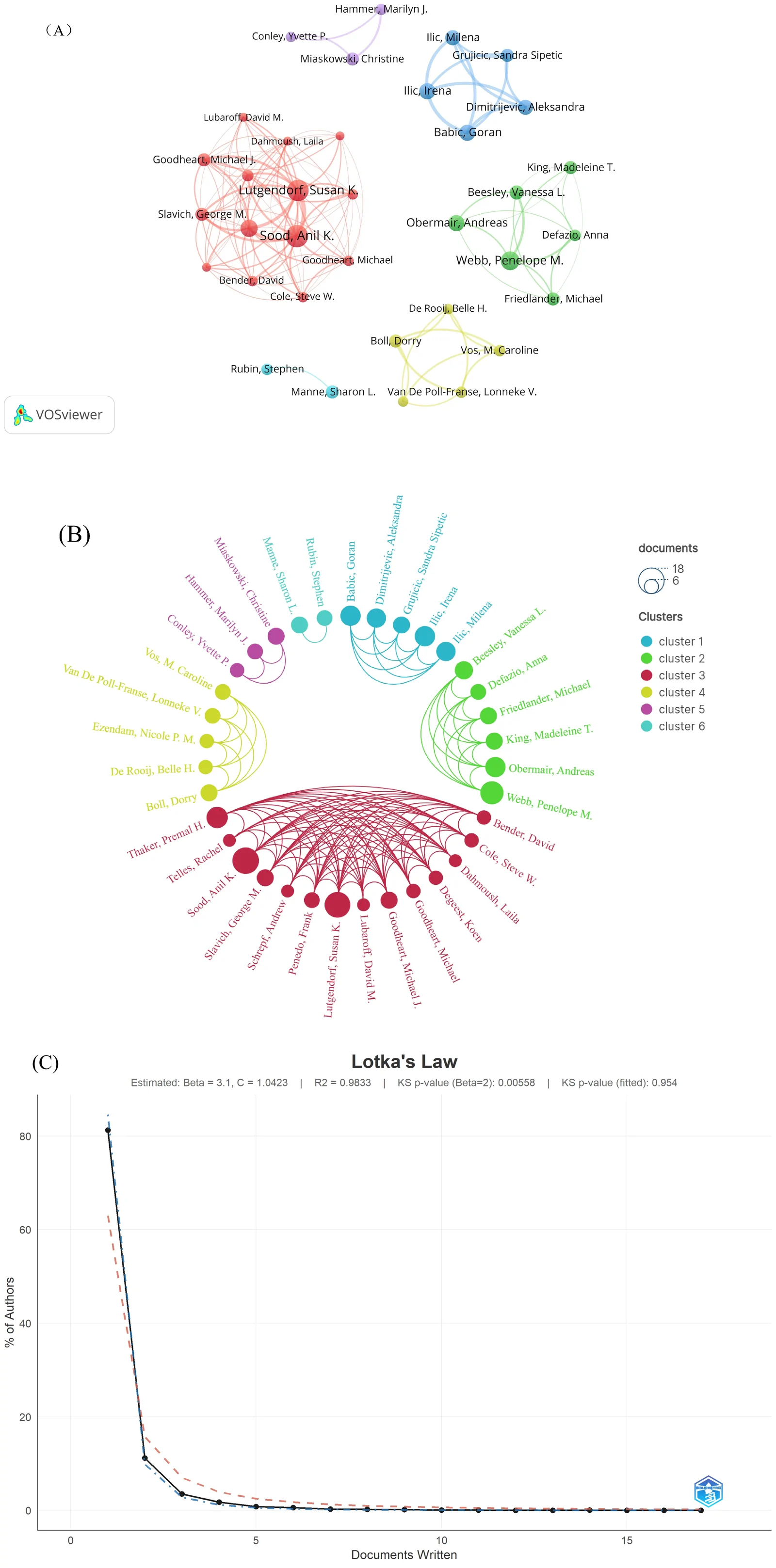
**(A)** Diagram of the collaborative network between authors; **(B)** collaborative chord chart between authors; **(C)** Lotka’s law analysis of author productivity.

Here, *N_max_* represents the number of publications by the most prolific author (37). Based on this formula and the maximum output of 17 publications, 35 core authors were identified. **Figure 5A** presents the collaboration network among the core authors and identifies six major clusters. Their connections are shown in the chord diagram in **Figure 5B**. **Table 3** presents the 10 most prolific authors. Sood, Anil K. and Lutgendorf, Susan K. ranked first and second, with 17 and 16 publications, respectively. Lutgendorf, Susan K. had the highest total citation count (865) and the highest average citation count per publication (54). The data indicate that the work of these authors has had a substantial impact on the field and has received considerable attention.

**Table 3:** The top 10 most productive authors.

Rank	Authors	Institution	Documents	Citations	Average citations
1	Sood, Anil K.	The University of Texas MD Anderson Cancer Center	17	858	50.47
2	Lutgendorf, Susan K.	University of Iowa	16	865	54.06
3	Webb, Penelope M.	QIMR Berghofer Medical Research Institute	13	499	38.38
4	Thaker, Premal H.	Washington University	11	331	30.09
5	Obermair, Andreas	Queensland Centre for Gynecological Cancer	10	210	21.00
6	Ilic, Irena	University of Belgrade	10	44	4.40
7	Babic, Goran	University of Kragujevac	10	44	4.40
8	Dimitrijevic, Aleksandra	University of Kragujevac	9	44	4.88
9	Ilic, Milena	University of Kragujevac	9	42	4.66
10	Beesley, Vanessa L.	University of Queensland	8	290	36.25

In the analysis based on Lotka’s law (**Figure 5C**), 81.3% of authors contributed a single publication. The fitted parameters were β = 3.10, C = 1.0423, and R^2^ = 0.9833. The software-reported Kolmogorov–Smirnov (KS) test indicated that the classical β = 2 form did not fit the observed distribution (p = 0.00558), whereas the distribution based on the fitted exponent was not rejected (p = 0.954).

Author-level analysis showed that although research on depression in gynecologic cancer has a certain level of activity, the structure of the research community remains relatively dispersed. According to Lotka’s law, related publications showed a typical long-tail distribution, meaning that most authors published only one paper, whereas a small number of authors accounted for the main academic output. Combined with the author collaboration network, this field appears to be driven mainly by a few sustained contributors, while a large number of authors participate only occasionally, suggesting that a stable and closely connected core author collaboration group has not yet formed.

### 3.5 Analysis of journals

**Figure 6:**
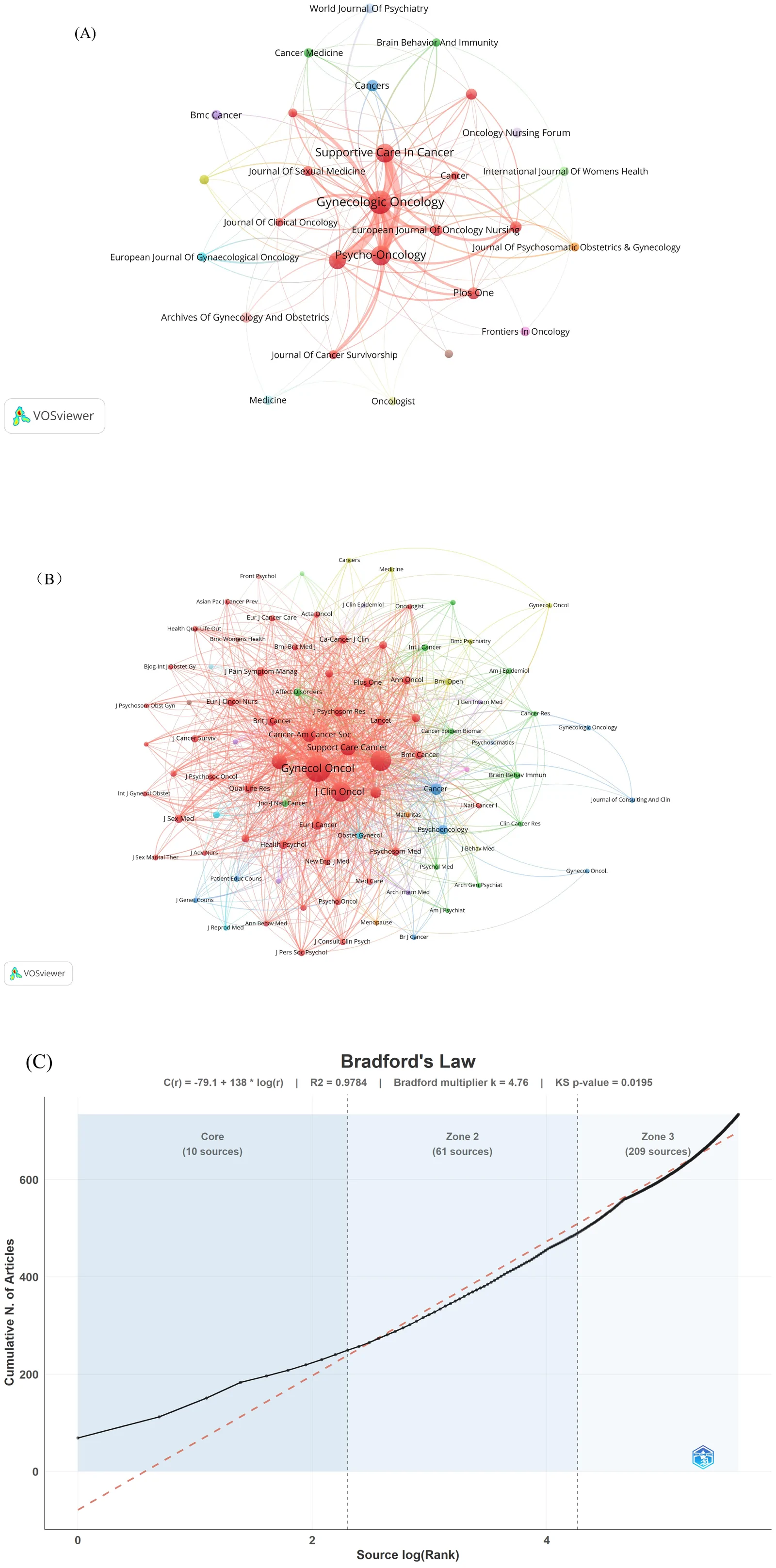
**(A)** Co-occurrence network diagram of the published journals; **(B)** co-occurrence network diagram of cited journals; **(C)** Bradford’s law analysis of publication sources.

The 734 publications appeared in 280 journals. **Table 4** lists the 10 journals with the highest publication counts, and **Figure 6A** displays the network of 28 journals that published at least five papers. *Gynecologic Oncology* ranked first with 69 publications, followed by *Psycho-Oncology* (n=43), *Supportive Care in Cancer* (n=39), and the *International Journal of Gynecological Cancer* (n=32). The United States accounted for the largest share of the top 10 journals, while the remaining journals were based in other high-income countries. No journal based in China appeared among the top 10. Most of these journals were classified as Q1 or Q2, although all had impact factors below 5.

**Table 4:** The top 10 most prolific journals.

Rank	Journal	Country	Counts	Division	IF (2024)
1	Gynecologic Oncology	USA	69	Q1	4.1
2	Psycho-Oncology	United Kingdom	43	Q2	3.5
3	Supportive Care in Cancer	USA	39	Q2	3
4	International Journal of Gynecological Cancer	USA	32	Q1	4.7
5	Plos One	USA	13	Q2	2.6
6	European Journal of Oncology Nursing	USA	12	Q1	2.7
7	Cancer Nursing	USA	11	Q1	2.5
8	Cancers	Switzerland	11	Q2	4.4
9	Asian Pacific Journal of Cancer Prevention	South Korea	10	Q3	2.514
10	Archives of Gynecology and Obstetrics	Germany	9	Q2	2.5

**Figure 6B** presents the co-occurrence network of 90 cited journals that received at least 50 citations. **Table 5** presents the 10 journals with the highest citation counts. *Gynecologic Oncology* was the most frequently cited journal (1,397 citations; IF = 4.1; Q1), followed by *Psycho-Oncology* (882 citations; IF = 3.5; Q2) and the *Journal of Clinical Oncology* (826 citations; IF = 43.4; Q1). All 10 journals were classified as Q1 or Q2, with Q1 journals accounting for 60%, indicating that these journals have substantial influence in the field of research on depression associated with gynecologic cancers.

**Table 5:** Top 10 co-citation journals.

Rank	Journal	Country	Citations	Division	IF (2024)
1	Gynecologic Oncology	USA	1397	Q1	4.1
2	Psycho-Oncology	United Kingdom	882	Q2	3.5
3	Journal of Clinical Oncology	USA	826	Q1	43.4
4	Cancer	USA	665	Q1	5.1
5	Support Care Cancer	USA	526	Q2	3
6	International Journal of Gynecological Cancer	USA	466	Q1	4.7
7	Cancer Nursing	USA	244	Q1	2.5
8	Journal of Psychosomatic Research	United Kingdom	204	Q2	3.3
9	European Journal of Cancer	United Kingdom	198	Q1	7.1
10	Plos One	USA	192	Q2	2.6

Analysis based on Bradford’s law (**Figure 6C**) showed that the 734 publications were distributed across 280 sources. The zoning analysis identified a 10-source core containing 249 publications (33.9%), a second zone comprising 61 sources and 241 publications (32.8%), and a third zone comprising 209 sources and 244 publications (33.2%). The cumulative relationship was fitted as C(r) = −79.1 + 138.0 ln(r), with R^2^ = 0.9784 and a Bradford multiplier of 4.76. Given the software-reported KS diagnostic (D = 0.1286, p = 0.0195), the observed pattern was characterized as approximate Bradford-like scattering rather than exact conformity to Bradford’s law.

Journal distribution and Bradford analysis indicated that research on depression in gynecologic cancer has a clear interdisciplinary nature, although publication output is mainly concentrated in a small number of core journals. Major sources such as Gynecologic Oncology, Psycho-Oncology, and Supportive Care in Cancer reflect the integration of gynecologic oncology, psycho-oncology, survivorship research, and supportive care in this field. This journal distribution pattern was consistent with the keyword clustering and co-citation results, suggesting that research on depression in gynecologic cancer is bringing cancer-specific clinical issues, psychological symptom burden, and quality-of-life outcomes into a shared research framework.

### 3.6 Analysis of cited references

**Figure 7:**
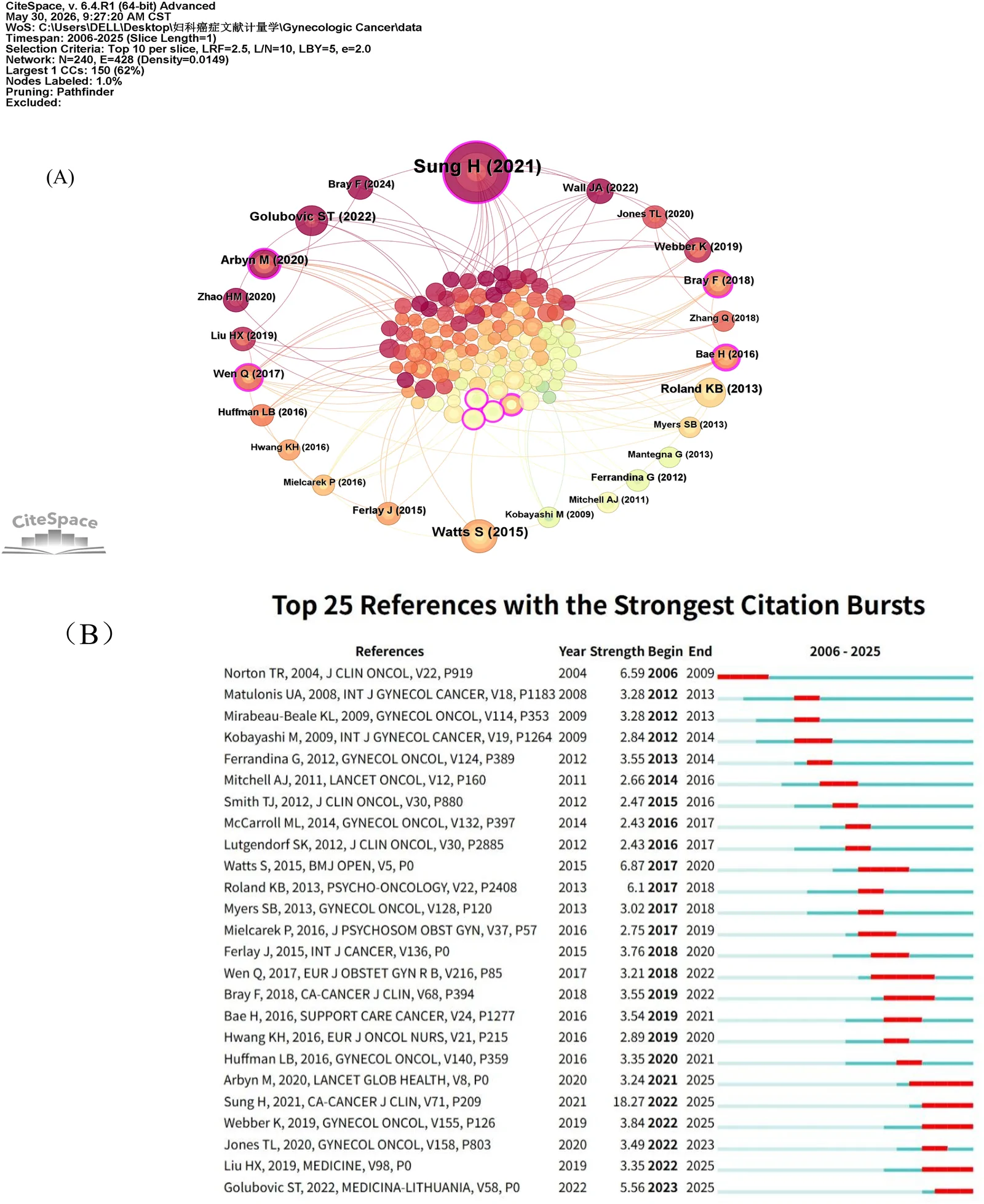
**(A)** Co-occurrence network diagram of cited references; **(B)** top 25 references with the strongest citation bursts.

Co-cited references refer to publications that are repeatedly cited together in subsequent articles. Owing to their high frequency of joint citation, these publications often constitute the knowledge base of a specific academic field (38). CiteSpace was used to construct a co-occurrence network of co-cited references (**Figure 7A**). The nodes represent cited references, and a larger node indicates that the reference has been cited more frequently. **Table 6** summarizes the 11 most frequently co-cited references, each receiving between 8 and 43 co-citations.

**Table 6:** The top 11 co-cited references.

Rank	Cited reference	Journal	First author	Citations	Year
1	Global Cancer Statistics 2020: GLOBOCAN Estimates of Incidence and Mortality Worldwide for 36 Cancers in 185 Countries.	CA Cancer J Clin	Sung H	43	2021
2	Depression and anxiety in ovarian cancer: a systematic review and meta-analysis of prevalence rates.	BMJ Open	Watts S	14	2015
3	Estimates of incidence and mortality of cervical cancer in 2018: a worldwide analysis	Lancet Glob Health	Arbyn M	12	2020
4	Risk Factors and Predictive Value of Depression and Anxiety in Cervical Cancer Patients	Medicina-Lithuania	Golubovic, ST	12	2022
5	Prevalence and predictors of psychological distress among women with ovarian cancer	Journal of Clinical Oncology	Norton TR	12	2004
6	A literature review of the social and psychological needs of ovarian cancer survivors	Psychooncology	Roland, KB	10	2013
7	Physical activity and exercise in women with ovarian cancer: A systematic review	Gynecologic Oncology	Jones, TL	8	2020
8	Global cancer statistics 2018: GLOBOCAN estimates of incidence and mortality worldwide for 36 cancers in 185 countries	CA Cancer J Clin	Bray F	8	2018
9	Mental distress, quality of life and social support in recurrent ovarian cancer patients during active chemotherapy.	Eur J Obstet Gynecol Reprod Biol	Wen, Q	8	2017
10	Moderate to severe distress in half of ovarian cancer patients undergoing treatment highlights a need for more proactive symptom and psychosocial management	Gynecologic Oncology	Wall JA	8	2022
11	OVQUEST - Life after the diagnosis and treatment of ovarian cancer - An international survey of symptoms and concerns in ovarian cancer survivor	Gynecologic Oncology	Webber, K	8	2019

**Figure 7B** shows the 25 references with the highest citation burst strengths, whose burst strengths ranged from 2.43 to 18.27. Citation bursts are indicated by red line segments, whose lengths are positively correlated with burst strength; that is, a longer segment indicates a stronger citation burst and a higher citation frequency (38). Notably, the strongest citation burst was observed for the study published by Sung H et al. in *CA: A Cancer Journal for Clinicians* in 2021, which quantified the global cancer burden (3). The longest citation burst occurred for the study by Arbyn M et al., published in *The Lancet Global Health* in 2020, on the incidence and mortality of cervical cancer (39). Additionally, five references exhibited citation bursts that continued through the end of the study period. Their themes were broadly consistent with those of the 11 most frequently co-cited references and were classified into three major categories: the epidemiology of cancer and gynecologic cancers, the quality of life of gynecologic cancer survivors, and factors associated with depression and its early screening in patients with gynecologic cancers.

Co-cited reference analysis provided an important knowledge base for interpreting the keyword clustering and burst results. Highly co-cited references and references with citation bursts were mainly concentrated on cancer epidemiology, quality of life among gynecologic cancer survivors, psychological distress, and depression screening. These findings were consistent with the keyword analysis, suggesting that research on depression in gynecologic cancer has formed a relatively stable conceptual core centered on depression, anxiety, quality of life, and major gynecologic cancer types, while gradually expanding toward survivorship care and mental health management.

### 3.7 Analysis of keywords

**Figure 8:**
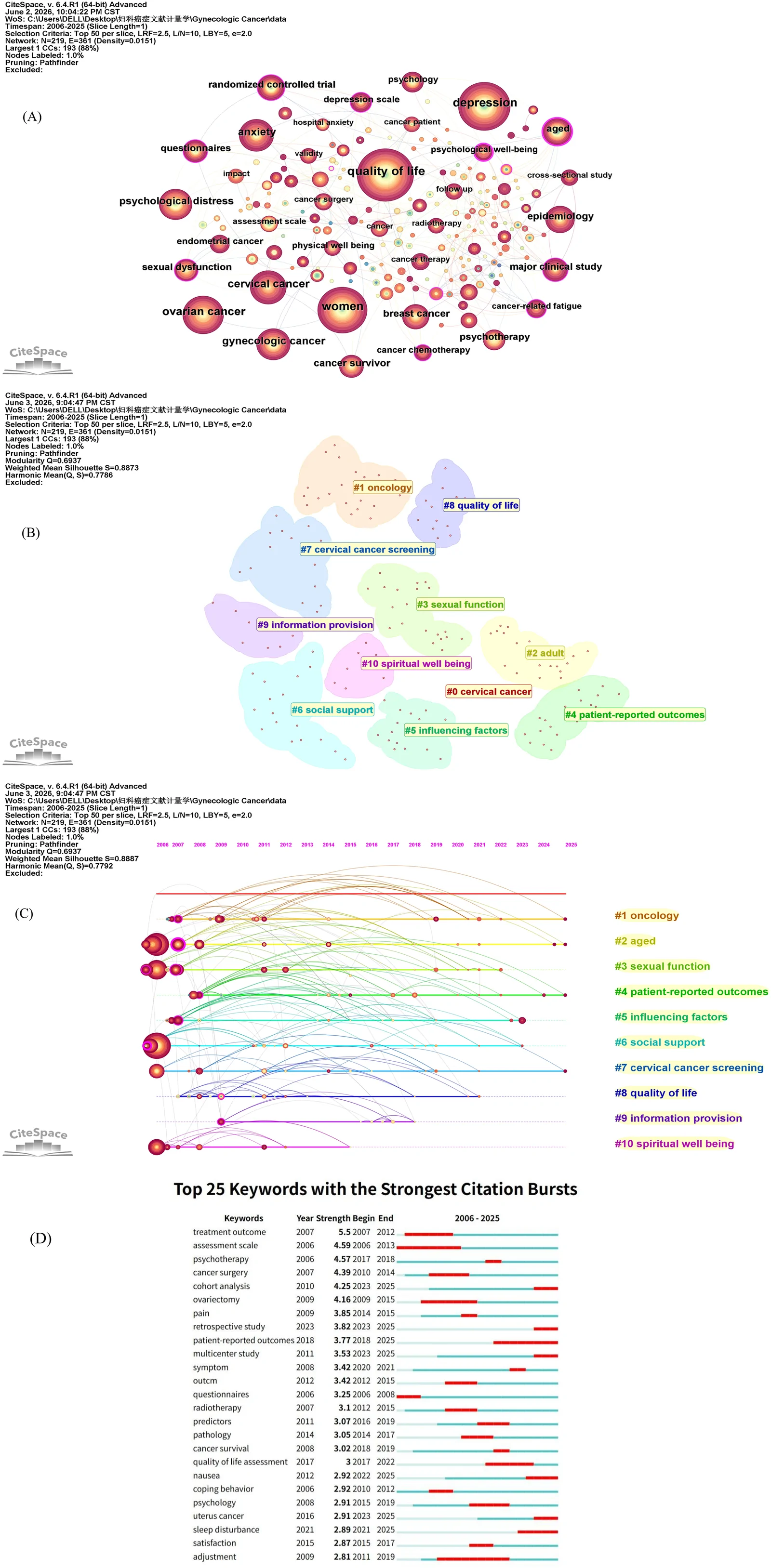
**(A)** Keyword co-occurrence mapping; **(B)** keyword clustering mapping; **(C)** keyword clustering timeline mapping; **(D)** keyword bursting mapping.

The 734 publications yielded 218 keywords, including 94 that appeared more than 10 times. The keyword co-occurrence network constructed using CiteSpace (**Figure 8A**) shows that node size represents keyword frequency. **Table 7** summarizes the 20 high-frequency keywords, each recorded more than 70 times. The two most frequent keywords were “quality of life” (n=400) and “depression” (n=346), followed by “women” (n=337), “ovarian cancer” (n=262), “cervical cancer” (206 occurrences), and “anxiety” (n=183). These findings closely aligned with the focus of this study. All 20 leading keywords were closely related to depression among patients with gynecologic cancers. The findings underscore the role of quality-of-life assessment in this field.

**Table 7:** Top 20 co-occurrence keywords involved.

Rank	Keyword	Counts	Rank	Keyword	Counts
1	quality of life	400	11	cancer survivor	111
2	depression	346	12	major clinical study	103
3	women	337	13	aged	101
4	ovarian cancer	262	14	randomized controlled trial	99
5	cervical cancer	206	15	sexual dysfunction	96
6	anxiety	183	16	questionnaires	95
7	gynecologic cancer	178	17	psychotherapy	93
8	psychological distress	161	18	endometrial cancer	77
9	epidemiology	125	19	depression scale	75
10	breast cancer	123	20	physical well being	73

The CiteSpace keyword map contained 11 clusters (**Figure 8B**). The modularity (Q = 0.6937) and silhouette (S = 0.7786) values supported the reliability of the clustering results. Cluster #0, “cervical cancer,” represented research on this cancer type. Clusters #6, “social support,” #9, “information provision,” and #10, “spiritual well-being,” covered factors related to depression. Clusters #8, “quality of life,” and #3, “sexual function,” addressed patient quality of life. Cluster #7, “cervical cancer screening,” focused on cervical cancer screening and the early identification of gynecologic cancers. Among the cancer types examined, cervical cancer received the greatest research attention. The keyword and co-cited reference analyses also identified treatment approaches and quality-of-life outcomes as major topics in research on gynecologic cancer-related depression. They also suggest growing academic attention to this issue and increasing efforts to develop interventions that alleviate symptoms and improve patients’ quality of life.

The keyword timeline map in **Figure 8C** shows how the research themes developed over time. The keywords were organized into 11 thematic clusters. Their temporal distribution indicates when these themes emerged and how long they remained active. **Figure 8C** shows that clusters #7, “cervical cancer screening,” #2, “aged,” #4, “patient-reported outcomes,” and #1, “oncology,” spanned nearly the entire timeline.

**Figure 8D** displays the 25 keywords with the highest burst strengths. Burst strength and duration were used to assess shifts in research attention over time. As shown in **Figure 8D**, “treatment outcome” had the strongest burst strength (5.5), whereas “adjustment” showed the longest burst period, spanning 9 years. In addition, “cohort analysis” (2023–2025), “retrospective study” (2023–2025), “patient-reported outcomes” (2018–2025), “nausea” (2022–2025), “multicenter study” (2023–2025), “uterus cancer” (2023–2025), and “sleep disturbance” (2021–2025) emerged frequently in recent years.

High-frequency keywords outlined the conceptual core of research on depression in gynecologic cancer, while keyword clusters and timeline maps further revealed the organizational relationships among different themes and their evolutionary trajectories. Recurring research themes included symptom burden and its measurement, cancer types and survivorship research, quality of life and psychosexual issues, supportive care needs, as well as interventions and outcome assessment. These findings were broadly consistent with the co-cited reference analysis and helped connect the visual knowledge maps with the substantive research content of the field.

### 3.8 Zipf-type distribution of keywords

The Zipf-type keyword rank-frequency analysis showed a strong descriptive log-log fit (log10[f(r)] = 3.661 − 1.464 log10(r), R^2^ = 0.9423; **Supplementary Figure 1**). The fitted exponent (s = 1.464) indicates a relatively steep decline in keyword frequency as rank increased, suggesting that this literature is organized around a small set of stable core concepts while retaining a long tail of more specific clinical, psychosocial, and methodological topics. Because visible deviations were observed among the highest-ranked keywords, this pattern was interpreted as approximate Zipf-type behavior rather than exact conformity to Zipf’s law.

### 3.9 Integrated thematic and temporal synthesis

**Figure 9:**
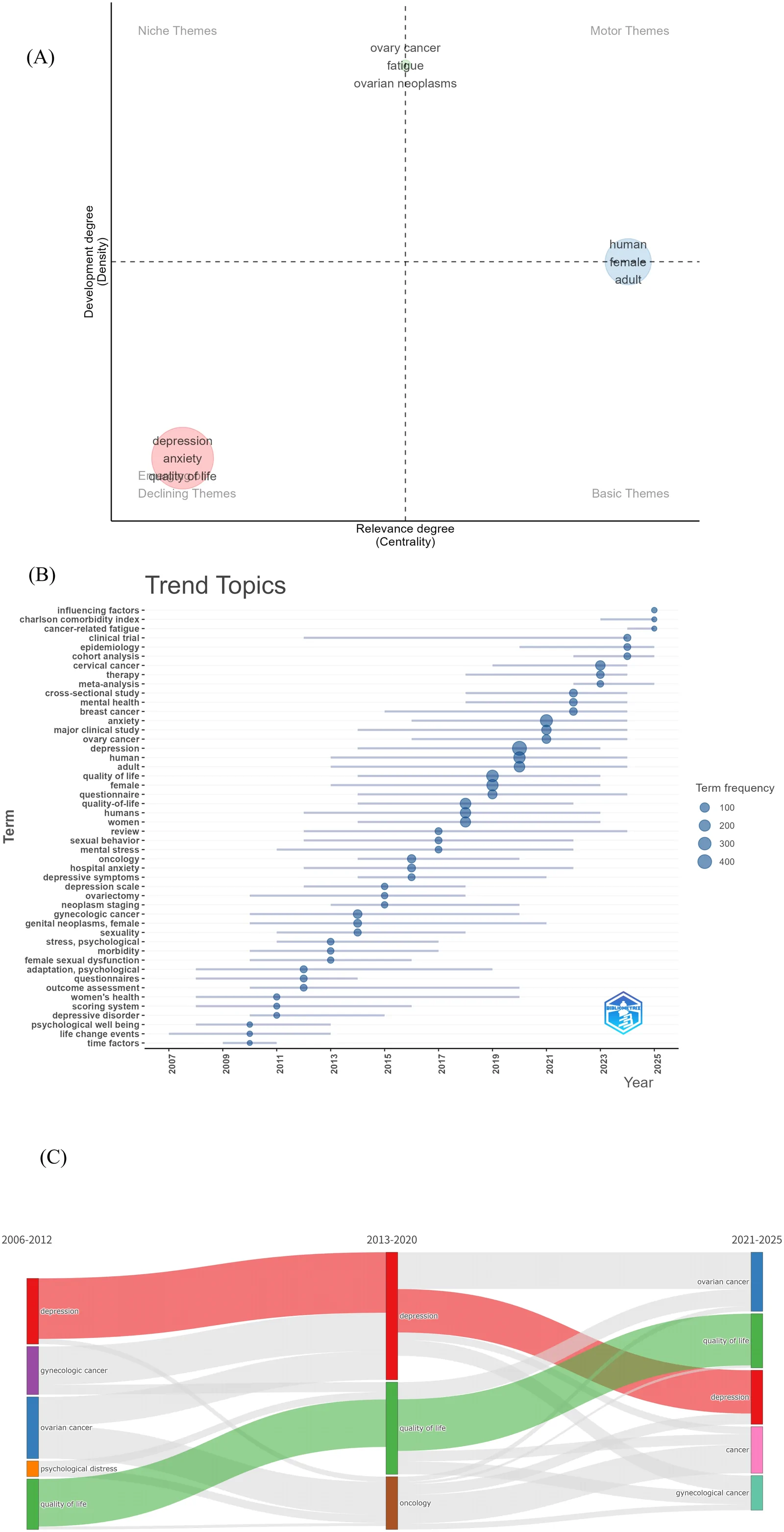
**(A)** Thematic map; **(B)** trend topics over time; **(C)** thematic evolution Sankey diagram.

Taken together, **Figures 8A, B**, thematic evolution (**Figure 9C**), trend topics (**Figure 9B**), and the thematic map (**Figure 9A**) indicate that research on depression in gynecologic cancer has formed an interconnected but internally heterogeneous knowledge structure. **Figure 9A** shows three relatively prominent co-word clusters: a depression-related thematic cluster centered on “depression,” “anxiety,” and “quality of life”; a clinical population descriptor cluster represented by “human,” “female,” and “adult”; and an ovarian cancer-related cluster associated with “fatigue” and “ovarian neoplasms.” It should be noted that the depression-related thematic cluster is located in the lower-left region of **Figure 9A** and should be interpreted cautiously in light of the current co-word analysis parameters. This position mainly reflects relatively low centrality and internal density, and does not indicate a decrease in research output or a decline in clinical importance. Similarly, the higher internal development of the ovarian cancer-related cluster should not be directly interpreted as indicating a greater disease burden or higher research quality.

Timeline mapping showed continued activity in clusters related to cervical cancer screening, “aged,” patient-reported outcomes, and oncology. Burst analysis also identified several terms that became more visible in recent years. “Patient-reported outcomes” showed a burst from 2018 to 2025; “sleep disturbance” from 2021 to 2025; “nausea” from 2022 to 2025; and “cohort analysis,” “retrospective study,” “multicenter study,” and the indexed term “uterus cancer” from 2023 to 2025. These patterns suggest growing attention to outcome reporting, study design, symptoms, and cancer sites. They should not be read as evidence that research on prevalence, distress, or quality of life has declined, because theme-specific frequencies were not compared across predefined periods. They also do not establish the clinical importance of these topics or the proportion of studies using each design. Similarly, absence from the leading keyword lists does not mean that a given population, cancer type, or topic has not been studied (25).

### 3.10 Historical development of research themes

**Figure 9C** showed a relatively clear pattern of continuity across different stages of research on depression in gynecologic cancer. The “depression” theme in 2006–2012 was connected to the theme with the same label in 2013–2020 with 25 links, sharing the terms “depression” and “anxiety.” During the same period, the “quality of life” theme continued into the next stage with 31 links, sharing terms including “quality of life,” “cervical cancer,” “gynecological cancer,” “emotional distress,” and “endometrial cancer.” These findings suggest that depression, anxiety, and quality of life constituted a persistent cross-period thematic framework in this field.

From 2013–2020 to 2021–2025, the strongest thematic continuity pathway was from “quality of life” to the theme with the same label, with 71 links. The “depression” theme was connected to the themes of “depression” and “ovarian cancer” with 54 and 51 links, respectively. Meanwhile, the connection between “quality of life” and “ovarian cancer” included terms such as “endometrial cancer,” “health-related quality of life,” and “patient-reported outcomes,” whereas its connection with “gynecological cancer” included “cancer survivors.” The connection from “depression” to “gynecological cancer” involved “fatigue” and “pain.” It should be noted that these values represent the number of links in the thematic evolution analysis rather than the number of publications corresponding to each theme.

Trend topic analysis further revealed the temporal distribution of related terms. “Anxiety” had a frequency of 261 and a median year of 2021. “Cervical cancer” and “therapy” both had a median year of 2023, with frequencies of 107 and 39, respectively. “Epidemiology” and “cohort analysis” both had a median year of 2024, with frequencies of 22 and 21, respectively. “Influencing factors,” “cancer-related fatigue,” and “Charlson comorbidity index” all had a median year of 2025, but their frequencies were only 6, 5, and 5, respectively. Because the first quartile years for “cancer-related fatigue” and “Charlson comorbidity index” were 2024 and 2023, respectively, a median year of 2025 does not mean that these terms first appeared in 2025. Considering their low frequencies, these terms are better interpreted as recently visible research signals rather than mature research frontiers.

Overall, thematic evolution in this field did not represent a simple replacement of early topics. Instead, persistent themes such as depression, anxiety, and quality of life continued to form new connections with more specific topics, including cancer types, accompanying symptoms, and patient-reported outcomes. The trend topic results also indicate that methodological terms such as epidemiology and cohort analysis have become more concentrated in recent years, suggesting that the field is gradually expanding from symptom description and quality-of-life assessment toward the identification of influencing factors and the accumulation of longitudinal evidence.

## 4 Discussion

### 4.1 General information

Research addressing depression in gynecologic cancer has expanded markedly over the past 20 years. Publication output increased steadily from 2006 to 2025, with particularly substantial growth after 2021. These findings indicate that this field is currently a major research hotspot.

The USA and China contributed the largest numbers of publications, well above the output of other countries and regions. Their leading positions may partly reflect the substantial burden of gynecologic cancer in both countries. In the USA, an estimated 110,000 new gynecologic cancer cases and 33,000 related deaths were projected for 2024 (40). In China, an estimated 590,000 new cases and 200,000 deaths related to gynecologic cancer occurred in 2022, indicating a substantial disease burden (41). These challenges may partly explain the sustained research attention and resource investment in China and the USA. Although China and the USA had the largest publication outputs, Canada recorded the highest mean citation count, at 36.4 citations per paper. By comparison, China averaged 12.6 citations per paper, ranking last among the 10 countries with the highest publication output. This indicates that although China has produced abundant research findings in this field, the quality and academic influence of its publications still need to be further improved.

The 12 institutions with the highest publication counts were mainly located in the USA and Australia, and more than half were based in the USA. The University of Texas MD Anderson Cancer Center ranked first in both publications and total citations, confirming the strong presence of US institutions in this area. This distribution may reflect differences in research funding, clinical resources, and health-system support. Although China ranked second in national publication output, only one Chinese institution appeared among the leading institutions, and its position was relatively low. This pattern may indicate that China’s research output is spread across a broader range of institutions rather than concentrated in a few leading centers. **Figure 4A** shows a certain degree of collaboration among these institutions; however, the depth and scope of collaboration remain limited. Future studies should strengthen interinstitutional collaboration and actively promote cross-regional cooperation to better investigate global research trends and hotspots in this field.

Publication output varied across countries and institutions, possibly because of differences in research funding and the ability to conduct clinical studies.

Among the core authors, Sood, Anil K. published the most papers (n=17), while Lutgendorf, Susan K. had the highest average citation count per publication. Although China ranked second in national publication output, no Chinese researcher appeared among the 10 most productive authors, suggesting that its research output was distributed across a wider group of researchers. The core authors formed six clusters, with frequent collaboration within clusters but fewer links between them. More cooperation across research teams could improve the sharing of data, resources, and expertise in gynecologic cancer-related depression research.

*Gynecologic Oncology*, *Psycho-Oncology*, and *Supportive Care in Cancer* published the largest numbers of articles and made substantial contributions to this field. Researchers working in this area may prioritize these journals when selecting potential publication venues. JCR quartile rankings and journal impact factors are commonly used to evaluate journal citation impact. Among the 10 most productive journals, all but one were classified as Q1 or Q2. However, their impact factors were all below 5, suggesting that although research on depression associated with gynecologic cancers is gaining attention and recognition, further high-quality studies are needed to attract support from journals with higher impact factors and enhance the influence of this field. Despite China’s high research output, no China-based journal appeared among the top 10. This limited representation suggests a need to strengthen the international visibility of journals from China and other parts of Asia.

Co-cited reference analysis was used to identify research priorities and trends in gynecologic cancer-related depression. The 10 most frequently co-cited references focused on depressive symptoms among patients with gynecologic cancers and the effects of cancer treatment on their psychological and emotional well-being. Watts S et al. conducted a meta-analysis of depression and anxiety in patients with ovarian cancer. They found that depression was approximately twice as prevalent in these patients as in women without a cancer diagnosis (9). Norton TR et al. assessed the mental health of 143 patients with ovarian cancer. More than half reported substantial distress related to the disease and its treatment, together with depressive symptoms. Many patients may have relied solely on psychotropic medications to alleviate emotional distress, while 60% did not use any mental health services (42). During the treatment of gynecologic cancers, attention to and intervention in patients’ mental health are as important as the management of their physical health. Future research and clinical practice should strengthen interdisciplinary collaboration and incorporate mental health interventions into comprehensive gynecologic cancer care to improve patients’ overall quality of life.

### 4.2 Research hotspots and trends

Based on the keyword and co-cited reference analyses, research on depression associated with gynecologic cancers can be categorized into four major areas: quality of life, depression associated with cervical cancer, sexual dysfunction, and the treatment of depression associated with gynecologic cancers. These areas capture the principal themes and recent shifts in scholarly attention.

#### 4.2.1 Quality of life

**Table 7** identifies “quality of life” as the most frequent keyword (n = 400). **Figure 8B** represents it as cluster #8, and **Figure 9C** shows that the quality-of-life theme had the strongest connection between 2013–2020 and 2021–2025, with 71 link occurrences and later terms related to patient-reported outcomes and cancer survivorship. These results identify quality of life as a persistent organizing theme, but they do not measure treatment effectiveness.

In recent years, technological advances and the emergence of novel therapies have improved the treatment of gynecologic cancers and significantly increased patient survival rates. Consequently, researchers have increasingly focused on the quality of life of patients with gynecologic cancers and survivors, while emphasizing the need for additional approaches to address quality-of-life issues throughout treatment (43, 44). Current evidence indicates that gynecologic cancers and their treatments can cause persistent or delayed health impairments, including bowel and urinary dysfunction, treatment-induced menopause and associated endocrine symptoms, sexual dysfunction, and impaired fertility (45). These problems are often accompanied by psychological distress and reduced social functioning, and their effects on the quality of life of patients and survivors may persist for years after treatment (45). Therefore, follow-up care and rehabilitation for gynecologic cancers should not be limited to the cancer itself but should also emphasize patients’ mental health and quality of life throughout diagnosis and treatment.

Quality of life is not only an important outcome measure reflecting patients’ subjective health experiences but may also have prognostic value. A study of patients with advanced epithelial ovarian cancer found that lower quality of life and deterioration in quality of life during follow-up led to shorter survival (46). Exercise may reduce depressive symptoms and fatigue while improving health-related quality of life in patients with gynecologic cancers (47). Evidence from a meta-analysis suggests that digital cognitive behavioral therapy and virtual reality-based interventions may lessen psychological distress and depressive symptoms while supporting better quality of life (48). This evidence supports routine quality-of-life assessment and appropriate supportive care for patients with gynecologic cancers throughout treatment and survivorship. In addition to depression, attention should be given to anxiety, fatigue, insomnia, sexual dysfunction, and other complications, with early interventions provided when necessary.

#### 4.2.2 Cervical cancer-related depression

**Table 7** recorded 206 occurrences of “cervical cancer,” fewer than “ovarian cancer” (n = 262), but sufficient to identify it as a prominent cancer-specific topic. **Figure 8B** includes cluster #0, labeled “cervical cancer,” while **Figure 8C** shows cluster #7, labeled “cervical cancer screening,” spanning nearly the entire timeline. In **Figure 9B**, “cervical cancer” had a trend-topic frequency of 107 and a median year of 2023. These mapping findings support a focused discussion of depression related to cervical cancer.

Data indicate that approximately 660,000 new cases of cervical cancer and 340,000 related deaths occur worldwide (49). Depression is estimated to affect approximately 38% of patients with cervical cancer (13). A cohort study involving 19,316 patients with cervical cancer further found that these patients had a significantly increased risk of subsequently developing depressive disorders compared with the non-cancer population (50). The diagnosis and treatment of cervical cancer not only impose physical burdens, such as pain and fatigue, but may also intensify psychological distress through hysterectomy- and chemoradiotherapy-induced changes in reproductive function, sexual dysfunction, and impaired body image. Lower educational attainment, financial strain, pain, and insufficient social support may contribute to more severe depressive symptoms (51). Among long-term survivors, financial distress, poor body image, lack of sexual activity, and lower existential well-being are also closely associated with depression (52).

Regarding treatment, clinical trials have shown that psychosocial telephone counseling can alleviate depression and cancer-related distress among cervical cancer survivors (53); nurse-led positive psychology interventions can reduce depressive symptoms while supporting sexual function and subjective well-being (54).

#### 4.2.3 Sexual dysfunction

**Table 7** shows that “sexual dysfunction” occurred 96 times. **Figure 8B** groups this term into cluster #3, labeled “sexual function,” which is positioned alongside cluster #8, labeled “quality of life.” **Figure 9C** further shows that “sexual function” was one of the shared terms in the strongest quality-of-life continuity pathway, which had 71 thematic link occurrences. These findings suggest that sexual health-related issues represent a research hotspot in this field.

Sexual dysfunction is a common but frequently overlooked long-term health problem among patients with gynecologic cancers and is closely associated with depressive symptoms and reduced quality of life (54). Gynecologic cancer surgery, chemotherapy, and pelvic radiotherapy can affect sexual response through alterations in reproductive anatomy, pelvic neurovascular injury, impaired ovarian function, and estrogen deficiency, often manifesting as reduced sexual desire, vaginal dryness, dyspareunia, difficulty reaching orgasm, and less frequent sexual activity (55). In women treated surgically for endometrial cancer, sexual function declines significantly during the early postoperative period. Poorer sexual function is significantly associated with impaired body image, reduced relationship quality, fear of sexual activity, and lower quality of life (56). Using DSM-5 diagnostic criteria, one study similarly found that 43.7% of gynecologic cancer survivors experienced sexual dysfunction accompanied by clinically significant distress, with sexual interest/arousal disorder being the most common (57).

Anhedonia, fatigue, and diminished interest caused by depression can reduce sexual desire, whereas dyspareunia, altered body image, impaired fertility, and difficulties in partner communication may exacerbate depression. A study involving 186 gynecologic cancer survivors showed that impaired sexual function was significantly associated with depressive symptoms, distress related to bodily changes, and reduced psychological quality of life (58). Clinical intervention studies further suggest that simultaneously addressing sexual health and psychological well-being may help improve the overall outcomes of patients with gynecologic cancers (54).

#### 4.2.4 Treatment and care of gynecologic cancer-related depression

Treatment and care for depression associated with gynecologic cancer also constitute an outcome-oriented and methodological strand of this field. In **Figure 8D**, “treatment outcome” had the strongest burst strength (5.5), “patient-reported outcomes” showed a burst from 2018 to 2025, and “cohort analysis,” “retrospective study,” and “multicenter study” each showed bursts from 2023 to 2025. **Figure 9B** further shows that “therapy” had a median year of 2023 and a frequency of 39. Overall, these findings suggest that recent bibliometric attention has been concentrated on treatment and care for depression associated with gynecologic cancer.

Psychological therapies can effectively alleviate symptoms such as depression and fatigue in patients with cancer (59). Cognitive behavioral intervention is one of the most extensively studied psychological treatments. It can correct negative cognitions in patients with gynecologic cancers and alleviate symptoms of depression, anxiety, fatigue, and distress (60). Studies have shown that chronic psychological stress can exacerbate inflammation, disrupt metabolism, and increase susceptibility to cancer. It can also suppress immune function, thereby promoting the growth of cancer cells (61). Cognitive behavioral interventions can alleviate anxiety and depression, indirectly reduce the immunosuppressive effects of chronic stress, and thereby improve the quality of life and prognosis of patients with gynecologic cancers. Given regional differences in demographic characteristics and cultural backgrounds, investigating psychotherapeutic approaches tailored to gynecologic cancer populations in different regions is essential. Cognitive behavioral stress management and biobehavioral interventions can also reduce anxiety, depressive symptoms, and fatigue in these patients (62, 63).

Psychotherapeutic interventions should incorporate family and social support, enhance patients’ coping capacity, improve their quality of life and treatment adherence, and be flexibly adapted according to the disease stage. During the early stages, preventive interventions such as cognitive behavioral therapy may be used to reduce the occurrence of depressive symptoms (64). During the advanced stages, greater emphasis should be placed on symptom relief and palliative psychological support to improve patients’ quality of life at the end of life (65).

### 4.3 Research directions

The keyword and co-cited reference analyses point to two areas that may receive greater attention in future work.

#### 4.3.1 Factors associated with depression in gynecologic cancers and early screening

The bibliometric findings support this research direction. Cluster #7 (“cervical cancer screening”) was visible across most of the keyword timeline, suggesting sustained attention to this topic. Among the 25 references with citation bursts, burst strength ranged from 2.43 to 18.27, and five bursts continued through 2025. These recent bursts included studies on factors associated with depression and early screening.

Depression associated with gynecologic cancers exhibits substantial individual variability. During treatment, the severity of patients’ depressive symptoms is associated with family income, cancer type, coping strategies, and personality traits (66). Prospective studies of patients with ovarian cancer have also shown that depression is not determined solely by tumor stage or treatment response; prior psychological status, personality traits, intrusive thoughts, and psychosocial resources also play important roles (67, 68). Therefore, future multicenter studies should encompass the stages of diagnosis, treatment, recurrence, survivorship, and palliative care, while comprehensively evaluating factors such as symptom burden, sexual dysfunction, impaired fertility, body image, fear of recurrence, pre-existing mental disorders, coping styles, financial stress, and social support. Moreover, general psychological distress should be distinguished from clinical depression using standardized depression scales, thereby providing a basis for the early identification and treatment of high-risk patients.

#### 4.3.2 Social support and supportive care

The keyword map showed separate clusters for social support (#6), information provision (#9), and spiritual well-being (#10), indicating that supportive care was reflected in several related strands rather than in a single theme. Recent bursts in “patient-reported outcomes” (2018–2025) and “multicenter study” (2023–2025) further point to increasing attention to measuring supportive needs and evaluating care in broader patient samples.

Social support and supportive care represent promising intervention targets in research on depression associated with gynecologic cancers. Social support enables patients with cancer to feel cared for and accepted by others (69). Clinical studies have shown that adequate support from family and friends, as well as emotional support, generally alleviates depressive symptoms (70). A longitudinal study of patients with gynecologic cancers undergoing chemotherapy found that social support significantly reduced their risk of depression (11). However, findings across studies have not been entirely consistent, suggesting that the source, type, quality, and timing of social support may be more important than the amount of support received (67, 68). Furthermore, symptom burden, financial difficulties, psychological distress, insufficient social support, and limited utilization of healthcare resources are associated with greater unmet care needs among patients with gynecologic cancers, particularly psychological, emotional, and health information needs (12).

Supportive care forms an integral part of cancer management. MASCC defines it as care directed toward preventing and managing the adverse effects of cancer and its treatment while addressing patients’ physical, psychological, and social needs across the disease course, from diagnosis and treatment to rehabilitation, survivorship, and end-of-life care (71, 72). In addition to treatment-related symptoms, patients with gynecologic cancers may experience sexual dysfunction, impaired fertility, fear of recurrence, and depression. Their illness experience may also affect partners and family caregivers, among whom greater unmet needs are closely associated with symptoms of depression and anxiety (73). Clinical studies have shown that caregivers’ unmet needs are primarily concentrated in psychological and emotional support, health information, and healthcare support. Moreover, greater psychological distress is associated with higher supportive care need (12, 74). Therefore, incorporating depression screening, symptom management, psychological interventions, and social resource support into routine clinical care may enhance treatment tolerability and continuity while supporting better psychological well-being and quality of life for patients. Initial clinical research in gynecologic oncology indicates that early palliative care can improve patients’ emotional well-being and quality of life (75). Notably, evidence specific to gynecologic cancers remains limited. Future research should further explore individualized, multidisciplinary supportive care models that span the entire disease trajectory.

#### 4.3.3 Implications for research and practice

From a research perspective, the integrated findings suggest several directions for future work. Prospective, multicenter studies would be useful, particularly when analyses are stratified by clinical factors such as cancer site and stage, treatment phase, and assessment time point. Studies should also report clearly which depression assessment instruments were used. Greater use of validated instruments, together with more consistent measurement and reporting of patient-reported outcomes, may make findings more comparable across studies.

From a practice perspective, the literature map produced in this study can help locate research on symptom burden, quality of life, psychosexual concerns, social support, sleep, interventions, and care services. However, it cannot determine how depression screening should be conducted or whether specific interventions are effective. Decisions about screening, referral, and intervention should still be based on clinical studies, systematic reviews, and relevant guidelines. The recurring themes identified here may therefore be used mainly to guide future research on clinical assessment and referral pathways.

**Figure 9C** showed a relatively clear pattern of continuity across different stages of research on depression in gynecologic cancer. The “depression” theme in 2006–2012 was connected to the theme with the same label in 2013–2020 with 25 links, sharing the terms “depression” and “anxiety.” During the same period, the “quality of life” theme continued into the next stage with 31 links, sharing terms including “quality of life,” “cervical cancer,” “gynecological cancer,” “emotional distress,” and “endometrial cancer.” These findings suggest that depression, anxiety, and quality of life constituted a persistent cross-period thematic framework in this field.

From 2013–2020 to 2021–2025, the strongest thematic continuity pathway was from “quality of life” to the theme with the same label, with 71 links. The “depression” theme was connected to the themes of “depression” and “ovarian cancer” with 54 and 51 links, respectively. Meanwhile, the connection between “quality of life” and “ovarian cancer” included terms such as “endometrial cancer,” “health-related quality of life,” and “patient-reported outcomes,” whereas its connection with “gynecological cancer” included “cancer survivors.” The connection from “depression” to “gynecological cancer” involved “fatigue” and “pain.” It should be noted that these values represent the number of links in the thematic evolution analysis rather than the number of publications corresponding to each theme.

Trend topic analysis further revealed the temporal distribution of related terms. “Anxiety” had a frequency of 261 and a median year of 2021. “Cervical cancer” and “therapy” both had a median year of 2023, with frequencies of 107 and 39, respectively. “Epidemiology” and “cohort analysis” both had a median year of 2024, with frequencies of 22 and 21, respectively. “Influencing factors,” “cancer-related fatigue,” and “Charlson comorbidity index” all had a median year of 2025, but their frequencies were only 6, 5, and 5, respectively. Because the first quartile years for “cancer-related fatigue” and “Charlson comorbidity index” were 2024 and 2023, respectively, a median year of 2025 does not mean that these terms first appeared in 2025. Considering their low frequencies, these terms are better interpreted as recently visible research signals rather than mature research frontiers.

Overall, thematic evolution in this field did not represent a simple replacement of early topics. Instead, persistent themes such as depression, anxiety, and quality of life continued to form new connections with more specific topics, including cancer types, accompanying symptoms, and patient-reported outcomes. The trend topic results also indicate that methodological terms such as epidemiology and cohort analysis have become more concentrated in recent years, suggesting that the field is gradually expanding from symptom description and quality-of-life assessment toward the identification of influencing factors and the accumulation of longitudinal evidence.

### 4.4 Limitations

Although we conducted an in-depth bibliometric analysis of research on depression associated with gynecologic cancers over the past 20 years and obtained relatively objective and comprehensive results, several limitations should be acknowledged. First, only English-language publications were included; therefore, some non-English publications may have been overlooked, although their overall number was relatively small. Second, the analysis covered only the past 20 years. Finally, recent studies may not be fully reflected because they have had less time to receive citations.

## 5 Conclusion

Data were obtained from the WOSCC, PubMed, and Scopus to investigate and evaluate global research output on depression associated with gynecologic cancers. The analysis also identified leading countries, institutions, and authors and mapped their collaboration networks. Publication output rose markedly over the past 20 years, indicating growing scholarly attention to depression associated with gynecologic cancers. The USA was the most productive country, and Sood, Anil K. was the most prolific author. *Gynecologic Oncology*, *Psycho-Oncology*, and Supportive Care in Cancer were the three most productive journals, whereas *Gynecologic Oncology*, *Psycho-Oncology*, and *Journal of Clinical Oncology* were the three most frequently cited journals. Current research primarily focuses on the quality of life and depression in patients with gynecologic cancers and survivors. Recent research trends include predictors of depression associated with gynecologic cancers and the treatment and care of this condition.
