# Supplementary material for: Research trends in depression associated with gynecologic cancers from 2006 to 2025: a multidatabase bibliometric and visual analysis
Source: Front Oncol. 2026 Aug 12;16:1910076. doi: 10.3389/fonc.2026.1910076 (PMC13507891; doi:10.3389/fonc.2026.1910076)
Supplement: Supplementary file 1 [file Table1.docx]

| Database | Search term |
| --- | --- |
| Web of  science | TS="Female Genital Neoplasm*") OR TS="Gynecologic Neoplasm*") OR TS="Gynecologic Tumor*") OR TS="Gynecologic Oncology*") OR TS="Gynecologic Cancer*") OR TS="Ovarian Neoplasm*") OR TS="Ovary Neoplasm*") OR TS="Ovary Cancer*") OR TS="Cancer* of Ovary") OR TS="Cancer* of the Ovary") OR TS="Ovarian Cancer*") OR TS="Ovarian Carcinoma*") OR TS="Ovary Carcinoma*") OR TS="Ovarian Tumor*") OR TS="Ovary Tumor*") OR TS="Cervical Neoplasm*,Uterine") OR TS="Cervix Neoplasm*") OR TS="Cervical Neoplasm*") OR TS="Cancer* of the Uterine Cervix") OR TS="Cancer* of Cervix") OR TS="Cancer* of the Cervix") OR TS="Cervix Cancer*") OR TS="Uterine Cervical Cancer*") OR TS=" Cervical Cancer*") OR TS="Cervix Carcinoma*") OR TS="Cervical Carcinoma*") OR TS="Cervix Tumor*") OR TS="Cervical Tumor*") OR TS="Endometrial Neoplasm*") OR TS="Endometrial Carcinoma*") OR TS="Cancer* of Endometrium") OR TS="Endometrium Cancer*") OR TS="Cancer* of the Endometrium") OR TS="Carcinoma* of Endometrium") OR TS="Endometrium Carcinoma*") OR TS="Endometrial Cancer*") OR TS="Endometrial Carcinoma*") OR TS="Endometrial Tumour*") OR TS="Endometrium Tumour*") OR TS="Uterine Sarcoma*") OR TS="Vulvar Neoplasm*") OR TS=" Vulva Neoplasm*") OR TS="Cancer* of Vulva") OR TS="Cancer* of the Vulva") OR TS="Vulvar Cancer*") OR TS="Vulva Cancer*") OR TS="Vulvar Carcinoma*") OR TS="Vulvar Tumour*") OR TS="Vulva Carcinoma*") OR TS="Vulva Tumour*") OR TS="Extramammary Paget* disease* of the vulva") OR TS="Vulvar Paget* disease*") OR TS="Vaginal Neoplasm*") OR TS="Vagina Neoplasm*") OR TS="Vaginal Cancer*") OR TS="Vagina Cancer*") OR TS="Cancer* of Vagina") OR TS="Cancer* of the Vagina" ) OR TS="Vaginal Carcinoma*") OR TS="Vaginal Tumour*") OR TS="Vagina Carcinoma*") OR TS="Vagina Tumour*") OR TS="Fallopian Tube Neoplasm*") OR TS="Fallopian Tube Cancer*") OR TS="Cancer*, Fallopian Tube") OR TS="Cancer* of the Fallopian Tube") OR TS="Fallopian Tube Carcinoma*") OR TS="Fallopian Tube Tumour*") OR TS="Primary peritoneal cancer*") OR TS="Primary peritoneal carcinoma*") OR TS="Gestational Trophoblastic Disease*") OR TS="Gestational Trophoblastic Neoplasia*") OR TS=" Gestational Trophoblastic Neoplasm*")) AND TS=Depression*) OR TS=Depressive*) OR TS=Depressed) OR TS=Despondent) OR TS=Melancholia*) OR TS="Depressive Symptom*") OR TS="Emotional Depression*") OR TS="Depressive Disorder*") OR TS="Depression* Disorder*") OR TS="Depressive Neurosis*") OR TS="Depression*, Endogenous") OR TS="Unipolar Depression") OR TS="Depressive Syndrome*") OR TS="Neurotic Depression*")) |
| PubMed | ("Female Genital Neoplasm*"[Title/Abstract] OR "Gynecologic Neoplasm*"[Title/Abstract] OR "Gynecologic Tumor*"[Title/Abstract] OR "Gynecologic Oncology*"[Title/Abstract] OR "Gynecologic Cancer*"[Title/Abstract] OR "Ovarian Neoplasm*"[Title/Abstract] OR "Ovary Neoplasm*"[Title/Abstract] OR "Ovary Cancer*"[Title/Abstract] OR "Cancer* of Ovary"[Title/Abstract] OR "Cancer* of the Ovary"[Title/Abstract] OR "Ovarian Cancer*"[Title/Abstract] OR "Ovarian Carcinoma*"[Title/Abstract] OR "Ovary Carcinoma*"[Title/Abstract] OR "Ovarian Tumor*"[Title/Abstract] OR "Ovary Tumor*"[Title/Abstract] OR "Cervical Neoplasm*,Uterine"[Title/Abstract] OR "Cervix Neoplasm*"[Title/Abstract] OR "Cervical Neoplasm*"[Title/Abstract] OR "Cancer* of the Uterine Cervix"[Title/Abstract] OR "Cancer* of Cervix"[Title/Abstract] OR "Cancer* of the Cervix"[Title/Abstract] OR "Cervix Cancer*"[Title/Abstract] OR "Uterine Cervical Cancer*"[Title/Abstract] OR " Cervical Cancer*"[Title/Abstract] OR "Cervix Carcinoma*"[Title/Abstract] OR "Cervical Carcinoma*"[Title/Abstract] OR "Cervix Tumor*"[Title/Abstract] OR "Cervical Tumor*"[Title/Abstract] OR "Endometrial Neoplasm*"[Title/Abstract] OR "Endometrial Carcinoma*"[Title/Abstract] OR "Cancer* of Endometrium"[Title/Abstract] OR "Endometrium Cancer*"[Title/Abstract] OR "Cancer* of the Endometrium"[Title/Abstract] OR "Carcinoma* of Endometrium"[Title/Abstract] OR "Endometrium Carcinoma*"[Title/Abstract] OR "Endometrial Cancer*"[Title/Abstract] OR "Endometrial Carcinoma*"[Title/Abstract] OR "Endometrial Tumour*"[Title/Abstract] OR "Endometrium Tumour*"[Title/Abstract] OR "Uterine Sarcoma*"[Title/Abstract] OR "Vulvar Neoplasm*"[Title/Abstract] OR " Vulva Neoplasm*"[Title/Abstract] OR "Cancer* of Vulva"[Title/Abstract] OR "Cancer* of the Vulva"[Title/Abstract] OR "Vulvar Cancer*"[Title/Abstract] OR "Vulva Cancer*"[Title/Abstract] OR "Vulvar Carcinoma*"[Title/Abstract] OR "Vulvar Tumour*"[Title/Abstract] OR "Vulva Carcinoma*"[Title/Abstract] OR "Vulva Tumour*"[Title/Abstract] OR "Extramammary Paget* disease* of the vulva"[Title/Abstract] OR "Vulvar Paget* disease*"[Title/Abstract] OR "Vaginal Neoplasm*"[Title/Abstract] OR "Vagina Neoplasm*"[Title/Abstract] OR "Vaginal Cancer*"[Title/Abstract] OR "Vagina Cancer*"[Title/Abstract] OR "Cancer* of Vagina"[Title/Abstract] OR "Cancer* of the Vagina" [Title/Abstract] OR "Vaginal Carcinoma*"[Title/Abstract] OR "Vaginal Tumour*"[Title/Abstract] OR "Vagina Carcinoma*"[Title/Abstract] OR "Vagina Tumour*"[Title/Abstract] OR "Fallopian Tube Neoplasm*"[Title/Abstract] OR "Fallopian Tube Cancer*"[Title/Abstract] OR "Cancer*, Fallopian Tube"[Title/Abstract] OR "Cancer* of the Fallopian Tube"[Title/Abstract] OR "Fallopian Tube Carcinoma*"[Title/Abstract] OR "Fallopian Tube Tumour*"[Title/Abstract] OR "Primary peritoneal cancer*"[Title/Abstract] OR "Primary peritoneal carcinoma*"[Title/Abstract] OR "Gestational Trophoblastic Disease*"[Title/Abstract] OR "Gestational Trophoblastic Neoplasia*"[Title/Abstract] OR " Gestational Trophoblastic Neoplasm*"[Title/Abstract]) AND (Depression*[Title/Abstract] OR Depressive*[Title/Abstract] OR Depressed[Title/Abstract] OR Despondent[Title/Abstract] OR Melancholia*[Title/Abstract] OR "Depressive Symptom*"[Title/Abstract] OR "Emotional Depression*"[Title/Abstract] OR "Depressive Disorder*"[Title/Abstract] OR "Depression* Disorder*"[Title/Abstract] OR "Depressive Neurosis*"[Title/Abstract] OR "Depression*, Endogenous"[Title/Abstract] OR "Unipolar Depression"[Title/Abstract] OR "Depressive Syndrome*"[Title/Abstract] OR "Neurotic Depression*"[Title/Abstract]) |
| Scopus | TITLE-ABS-KEY"Female Genital Neoplasm*") OR TITLE-ABS-KEY"Gynecologic Neoplasm*") OR TITLE-ABS-KEY"Gynecologic Tumor*") OR TITLE-ABS-KEY"Gynecologic Oncology*") OR TITLE-ABS-KEY"Gynecologic Cancer*") OR TITLE-ABS-KEY"Ovarian Neoplasm*") OR TITLE-ABS-KEY"Ovary Neoplasm*") OR TITLE-ABS-KEY"Ovary Cancer*") OR TITLE-ABS-KEY"Cancer* of Ovary") OR TITLE-ABS-KEY"Cancer* of the Ovary") OR TITLE-ABS-KEY"Ovarian Cancer*") OR TITLE-ABS-KEY"Ovarian Carcinoma*") OR TITLE-ABS-KEY"Ovary Carcinoma*") OR TITLE-ABS-KEY"Ovarian Tumor*") OR TITLE-ABS-KEY"Ovary Tumor*") OR TITLE-ABS-KEY"Cervical Neoplasm*,Uterine") OR TITLE-ABS-KEY"Cervix Neoplasm*") OR TITLE-ABS-KEY"Cervical Neoplasm*") OR TITLE-ABS-KEY"Cancer* of the Uterine Cervix") OR TITLE-ABS-KEY"Cancer* of Cervix") OR TITLE-ABS-KEY"Cancer* of the Cervix") OR TITLE-ABS-KEY"Cervix Cancer*") OR TITLE-ABS-KEY"Uterine Cervical Cancer*") OR TITLE-ABS-KEY" Cervical Cancer*") OR TITLE-ABS-KEY"Cervix Carcinoma*") OR TITLE-ABS-KEY"Cervical Carcinoma*") OR TITLE-ABS-KEY"Cervix Tumor*") OR TITLE-ABS-KEY"Cervical Tumor*") OR TITLE-ABS-KEY"Endometrial Neoplasm*") OR TITLE-ABS-KEY"Endometrial Carcinoma*") OR TITLE-ABS-KEY"Cancer* of Endometrium") OR TITLE-ABS-KEY"Endometrium Cancer*") OR TITLE-ABS-KEY"Cancer* of the Endometrium") OR TITLE-ABS-KEY"Carcinoma* of Endometrium") OR TITLE-ABS-KEY"Endometrium Carcinoma*") OR TITLE-ABS-KEY"Endometrial Cancer*") OR TITLE-ABS-KEY"Endometrial Carcinoma*") OR TITLE-ABS-KEY"Endometrial Tumour*") OR TITLE-ABS-KEY"Endometrium Tumour*") OR TITLE-ABS-KEY"Uterine Sarcoma*") OR TITLE-ABS-KEY"Vulvar Neoplasm*") OR TITLE-ABS-KEY" Vulva Neoplasm*") OR TITLE-ABS-KEY"Cancer* of Vulva") OR TITLE-ABS-KEY"Cancer* of the Vulva") OR TITLE-ABS-KEY"Vulvar Cancer*") OR TITLE-ABS-KEY"Vulva Cancer*") OR TITLE-ABS-KEY"Vulvar Carcinoma*") OR TITLE-ABS-KEY"Vulvar Tumour*") OR TITLE-ABS-KEY"Vulva Carcinoma*") OR TITLE-ABS-KEY"Vulva Tumour*") OR TITLE-ABS-KEY"Extramammary Paget* disease* of the vulva") OR TITLE-ABS-KEY"Vulvar Paget* disease*") OR TITLE-ABS-KEY"Vaginal Neoplasm*") OR TITLE-ABS-KEY"Vagina Neoplasm*") OR TITLE-ABS-KEY"Vaginal Cancer*") OR TITLE-ABS-KEY"Vagina Cancer*") OR TITLE-ABS-KEY"Cancer* of Vagina") OR TITLE-ABS-KEY"Cancer* of the Vagina" ) OR TITLE-ABS-KEY"Vaginal Carcinoma*") OR TITLE-ABS-KEY"Vaginal Tumour*") OR TITLE-ABS-KEY"Vagina Carcinoma*") OR TITLE-ABS-KEY"Vagina Tumour*") OR TITLE-ABS-KEY"Fallopian Tube Neoplasm*") OR TITLE-ABS-KEY"Fallopian Tube Cancer*") OR TITLE-ABS-KEY"Cancer*, Fallopian Tube") OR TITLE-ABS-KEY"Cancer* of the Fallopian Tube") OR TITLE-ABS-KEY"Fallopian Tube Carcinoma*") OR TITLE-ABS-KEY"Fallopian Tube Tumour*") OR TITLE-ABS-KEY"Primary peritoneal cancer*") OR TITLE-ABS-KEY"Primary peritoneal carcinoma*") OR TITLE-ABS-KEY"Gestational Trophoblastic Disease*") OR TITLE-ABS-KEY"Gestational Trophoblastic Neoplasia*") OR TITLE-ABS-KEY" Gestational Trophoblastic Neoplasm*")) AND TITLE-ABS-KEYDepression*) OR TITLE-ABS-KEYDepressive*) OR TITLE-ABS-KEYDepressed) OR TITLE-ABS-KEYDespondent) OR TITLE-ABS-KEYMelancholia*) OR TITLE-ABS-KEY"Depressive Symptom*") OR TITLE-ABS-KEY"Emotional Depression*") OR TITLE-ABS-KEY"Depressive Disorder*") OR TITLE-ABS-KEY"Depression* Disorder*") OR TITLE-ABS-KEY"Depressive Neurosis*") OR TITLE-ABS-KEY"Depression*, Endogenous") OR TITLE-ABS-KEY"Unipolar Depression") OR TITLE-ABS-KEY"Depressive Syndrome*") OR TITLE-ABS-KEY"Neurotic Depression*")) |
